# *“I always find myself very tired and exhausted”*: The physical impact of caring; a descriptive phenomenological study of the experiences of prostate cancer caregivers in Cape Coast, Ghana

**DOI:** 10.1371/journal.pone.0268627

**Published:** 2022-07-26

**Authors:** Benedicta Owoo, Jerry Paul K. Ninnoni, Evelyn Asamoah Ampofo, Abdul-Aziz Seidu

**Affiliations:** 1 School of Nursing and Midwifery, University of Cape Coast, Cape Coast, Ghana; 2 Centre for Gender and Advocacy, Takoradi Technical University, Takoradi, Ghana; 3 College of Public Health, Medical and Veterinary Sciences, James Cook University, Townsville, Australia; Universidade Estadual Paulista Julio de Mesquita Filho Faculdade de Medicina Campus de Botucatu, BRAZIL

## Abstract

**Introduction:**

Prostate cancer is a significant public health burden and a significant cause of morbidity and mortality among men worldwide. This study, therefore, explored how caring affects the physical health of family caregivers of prostate cancer patients.

**Method:**

The study adopted a descriptive phenomenological method. Twelve participants were recruited using the purposive sampling technique. A semi-structured face to face, in-depth interviews were conducted with family caregivers of patients living with prostate cancer. The interviews were transcribed verbatim, and the data were analysed using Colaizzi’s phenomenological approach.

**Findings:**

The family caregiver’s experience with the physical impact associated with caregiving uncovered two significant themes with six sub-themes. “Rest and Sleep” emerged as the first central theme, with sleeplessness, fatigue, pain, and worsening pre-existing conditions as the sub-themes. The second main theme was ‘Nutrition’ with altered eating patterns and weight loss emerging as sub-themes.

**Conclusion:**

The study suggests that family caregivers of patients treated for prostate cancer may struggle with physical consequences associated with the caregiving role, which impacts their physical health. It is of great importance, especially for nurses, to come up with measures to minimise these adverse physical effects on the family caregivers through formal education programmes and training on how to care for these patients at home.

## Introduction

Globally, more than 10 million new cancer cases are reported annually, according to the World Health Organization [WHO] [1]. It is estimated that 9.6 million deaths were attributed to cancer in 2018, with 506 000 deaths occurring in sub-Saharan Africa 2018 [1]. Prostate cancer is a significant public health burden and a substantial cause of morbidity and mortality among men worldwide [2]. Among cancers, prostate cancer is the second most common cancer affecting men globally [2]. The American Institute for Cancer Research [AICR] report showed 1.3 million new cases recorded in 2018 [3].

Reports have shown that prostate cancer is the most commonly diagnosed cancer in Ghana and the main contributor to cancer-related deaths among men [4]. High mortality rates have been attributed to late presentation and lack of screening for Prostate-specific antigen (PSA) due to limited access to healthcare, culture and ignorance [5]. Thus, a low detection rate, poor management, and increased mortality of 75% in men [5], with only a 17.7% survival rate [6]. The disease burden is immense, not only for the affected individuals but also for their relatives and friends. Family caregivers unavoidably become the long-term care providers of these patients with little or no preparation, support and guidance from healthcare providers.

Caring for a patient with prostate cancer is very demanding and puts much stress on caregivers. The family caregivers, such as spouses, children, relatives, friends, and loved ones, support activities of daily living [7]. Family caregivers are unpaid and have no formal training to provide these services and, therefore, are faced with many challenges, including impacts on their physical well-being [8]. Physical challenges reported by family caregivers include; sleep disturbance, fatigue, pain, loss of physical strength, loss of appetite, and weight loss [9]. In an Australian study of caregivers, more than half reported that caregiving had directly affected their overall physical health [10], including tiredness and exhaustion 54.5%; back, neck, and shoulder problems 33.8%; blood pressure and heart problems 12.6%; arthritis 10%; stress-related illnesses 6.6%; being physically unfit and weight problems 5.5%; issues of digestion and bowel 4.6%; and leg and foot problems 4.6%. Briggs and Fisher [10] studied caregivers of people with advanced cancer. They found that more than two-thirds reported fatigue of 69% at baseline, which increased as time went on, resulting in patient deterioration [11]. Fatigue decreased the ability to concentrate in 69% of cancer caregivers, reduced motivation in 58%, affected relationships in 46%, reduced ability to perform usual activities in 42%, and affected mood in 35% [12].

Similarly, a study conducted in China among cancer patients’ spouse’s by Yao, Guo, Yuan and Zhang [13] revealed that Chinese cancer patients’ spouses experienced higher levels of fatigue symptoms associated with critical caregiving-related factors. Medical expenses, education level, family income, support from other family members, caregiving time, and coping styles. Grbich et al. [11] also identified a substantial report on the physical impact of the caring role. In their report, caregivers stated that heavy lifting caused back and leg strain, with further physical strain when the patient was incontinent with urine and faeces due to the increased amount of washing.

A continual lack of sleep exacerbated physical well being if patients needed care during the night. Up to 82% of female cancer caregivers reported sleep disturbances, with a significant number associated with depression, anger, and anxiety [14], due to continuous worrying about the patient’s health [15]. On the other hand, Given et al. [45] reported that caregivers experience headaches and severe body aches due to the caregiving role in their study on support for caregivers of cancer patients. The literature suggests that a higher caregiver burden is associated with an increased caregiver mortality rate [16]. Vedhara and Irwin [17] also posit that adverse health effects of caring for a person with prostate cancer are caused by the lack of time to devote to self-care and negligence of protective health behaviours (such as maintaining a healthy diet, exercising routinely, keeping medical appointments, and getting adequate sleep) due to the caregiving burden.

On the contrary, while some family caregivers considered the caring role a burden, others believe that the position provided positive emotions or high satisfaction. Time spent together was described as “Precious time”, which allowed for the expression of their love for the patient [18]. This assertion is supported by Akpan-idiok and Anarado’s [19] study on the perception of caregiving benefits to patients with advanced cancer. In their findings, family caregivers reported that caring for a patient with advanced cancer was rewarding. It provided satisfaction, closeness with the advanced cancer patient and a sense of fulfilment. According to Gonzalez, Roman-Calderon, and Limonero [22], this enabled the caregivers to perceive the role as less stressful, hence, the active participation in care.

Evidence, however, suggests that several studies have been conducted in the area of prostate cancer in Ghana [19–25]. Nevertheless, most of these studies focused exclusively on improving patient outcomes (e.g management, treatment, prevalence, awareness, knowledge and attitude). For example, Ofori [29] explored the experience of caregivers of patients with prostate cancer, but the study was limited and focused on spousal experience. Yet, little is known about the physical impact of the caregiving role on the family caregivers of patients with prostate cancer within the Cape Coast Metropolis. Also, per the evidence available, no study has been conducted in this area within the Cape Coast Metropolis, the largest referral hospital in Central Ghana. Therefore, having a better understanding of the family caregiver’s critical roles and the impact of caring on their physical well-being may assist health care professionals, especially nurses, by supporting caregivers with tasks, services, training, resources and interventions towards meeting these needs and thus improving caregiver quality of life.

## Materials and methods

### Study design

The study adopted a qualitative phenomenological study design grounded in the descriptivist research paradigm. A qualitative phenomenology approach allows researchers to put aside their perceptions of a phenomenon and give meaning to participants’ experiences [26]. It focuses on the lived experience within a particular group, and its fundamental goal is to arrive at a description of a specific field of the phenomenon [27]. Hence, the selection of descriptive phenomenology as the methodology of choice to explore the lived experiences of family caregivers of patients with prostate cancer. A semi-structured face-to-face interview was used to elicit information on the physical impact of caring for patients with prostate cancer among family caregivers within the Cape Coast metropolis of Ghana. The ultimate goal of this study design is to put the study in a social context and understand the findings from the participants’ subjective experiences [28]. A purposive sampling technique was adopted in selecting the participants. This technique was chosen to select participants who know about the phenomena of caring for patients with prostate cancer. The consolidated criteria for reporting qualitative research (COREQ) was adopted in reporting this research (S1 Appendix).

### Study setting

The study was conducted at the Genito-urinary unit of the Cape Coast Teaching Hospital. The Cape Coast Teaching Hospital is one of Ghana’s three most prominent hospitals. The hospital receives many referral cases covering the central part of Ghana and beyond. It serves medical and allied health students from the University of Cape Coast and is a learning centre for several health professionals. The hospital has several units, including a Genito-urinary team to manage cancer cases. This facility was selected because the Central region has no other hospital that cares for prostate cancer patients apart from the Cape Coast Teaching Hospital. Also, per the researchers’ knowledge based on a literature review, there is little study on prostate cancer and the impact of caregiving on family caregivers undertaken in the Central region of Ghana.

### Pretesting of interview guide

Pretesting allows the researcher to amend possible errors before applying them to the actual sample [29]. It was conducted with two participants at the Cape Coast Teaching Hospital. The interview guide was divided into three sections. Section A was on the participants’ demographic data (age, sex, highest educational level, occupation, religion, and the relationship with the patient). Section B explored family caregivers’ experiences of caring for patients with prostate cancer (Physical impact). Section C explored any other areas that the participant may want to discuss that may not have been addressed. Previous literature guided the development of the interview guide, using open-ended questions with probes. Two questions under section B of the guide were revised after the pre-testing. The question merged into the other was, “Do you know the name of cancer your relative is suffering from?” It appears unclear to participants, and this was revised to read “Please kindly tell me about your relation’s condition” (S2 Appendix).

### Participants recruitment

Following ethical clearance, a meeting was held with the Genito-urinary Unit manager to explain the study further and discuss recruitment strategies as outlined in the study protocol. Initial contact was made with the prostate cancer patients in the consulting room of the Genito-urinary unit. The doctors identified those patients who met the inclusion criteria (Patients diagnosed with prostate cancer but not Benign Prostatic Hyperplasia) and informed them about the research. These patients were introduced to the lead author (BO conducted the interviews), who provided details of the study and recruited those willing to be part. They were asked to identify their family caregivers, and their contact information was retrieved. The lead researcher contacted the family caregivers on the telephone and explained the importance of the study. Twelve participants were recruited for the study. None of the family caregivers declined.

### Inclusion criteria and exclusion criteria

As part of the inclusion criteria for the study, family caregivers who provided the most assistance to prostate cancer patients were selected. Spouse, children, parents, relatives, friends and loved ones involved in the care of prostate cancer patients for at least six months and above, and those aged ≥ 18 years were selected. The study, however, excluded bereaved family caregivers who no longer cared for patients living with prostate cancer. Family caregivers who had less than six months of experience and less than 18 years were also excluded. Patients with enlarged prostate glands suggestive of Benign Prostatic Hyperplasia were also excluded from the study.

### Data collection process

The interview was scheduled and conducted at agreed venues, dates, and times. The interviews were semi-structured face-to-face. Some interviews were conducted at the participant’s home and others within the hospital premises. The interview involved just the researcher and the participant; no one else was present. Each participant was given a consent form to read and sign/thumbprint. Those who did not have the literacy ability were assisted in ensuring they provided informed consent. In instances where participants could not read English, the researcher translated it into the local language (Twi) to ensure they understood everything written on the consent form. Participants were assured that the interview was for research purposes. The data collection lasted between March and April 2019.

All interviews were audio-taped with participant consent and then later transcribed. During the interviews, there was probing and redirecting of responses when necessary to focus on the discussion and get in-depth and rich reactions from participants. The nonverbal communications of participants, such as body language and mannerisms, were recorded in a field diary which helped during transcription. Participants were informed about further interview sessions or phone calls for clarification when necessary. The goal at this point was to attain ‘saturation’ as emphasised by Glaser and Strauss [30]. Creswell [31] nevertheless recommends 5–25 participants. Therefore, Creswell’s [31] and Glaser and Strauss’s [30] recommendations were considered to determine saturation. The interviews were ‘saturated’ with twelve participants. No new information was discovered, thus signalling that data collection may not add new meaning [32]. Transcription was done concurrently with the interview. On average interview lasted 47 minutes.

### Data analyses

The taped caregiver interviews were transcribed verbatim by the lead researcher. The lead researcher (BO), a female, has a Master of Nursing degree and has attended several workshops on conducting qualitative research. Data were analysed using a thematic analysis approach consistent with the seven steps outlined by Colaizzi [33] for qualitative data analysis. Data analysis was concurrent with data collection.

The ***first step in data analysis involved reading and rereading all participants’ verbatim transcripts to be familiarised with the data***. By so doing, a deeper understanding and a sense of ‘feel’ for what was described by the participants was achieved by the researchers by reading through the transcripts several times. ***The second step involved extracting significant statements***. *The researchers (BO*, *JPN*, *EAA) identified and removed substantial statements and phrases from the transcribed document at this analysis stage*. JPN and EAA are experienced in qualitative research and have published numerous qualitative research articles. By the end of step two, a list of significant statements related directly to the study (codes) (example: loss of appetite, constantly tired, sudden loss of weight etc.) had been extracted from the transcribed data. All researchers compared removed statements, and in the end, a consensus was reached. ***Step three involved understanding the meaning behind each significant statement/phrase by creating a formulated meaning unit for each big statement***.

**Fig 1:**
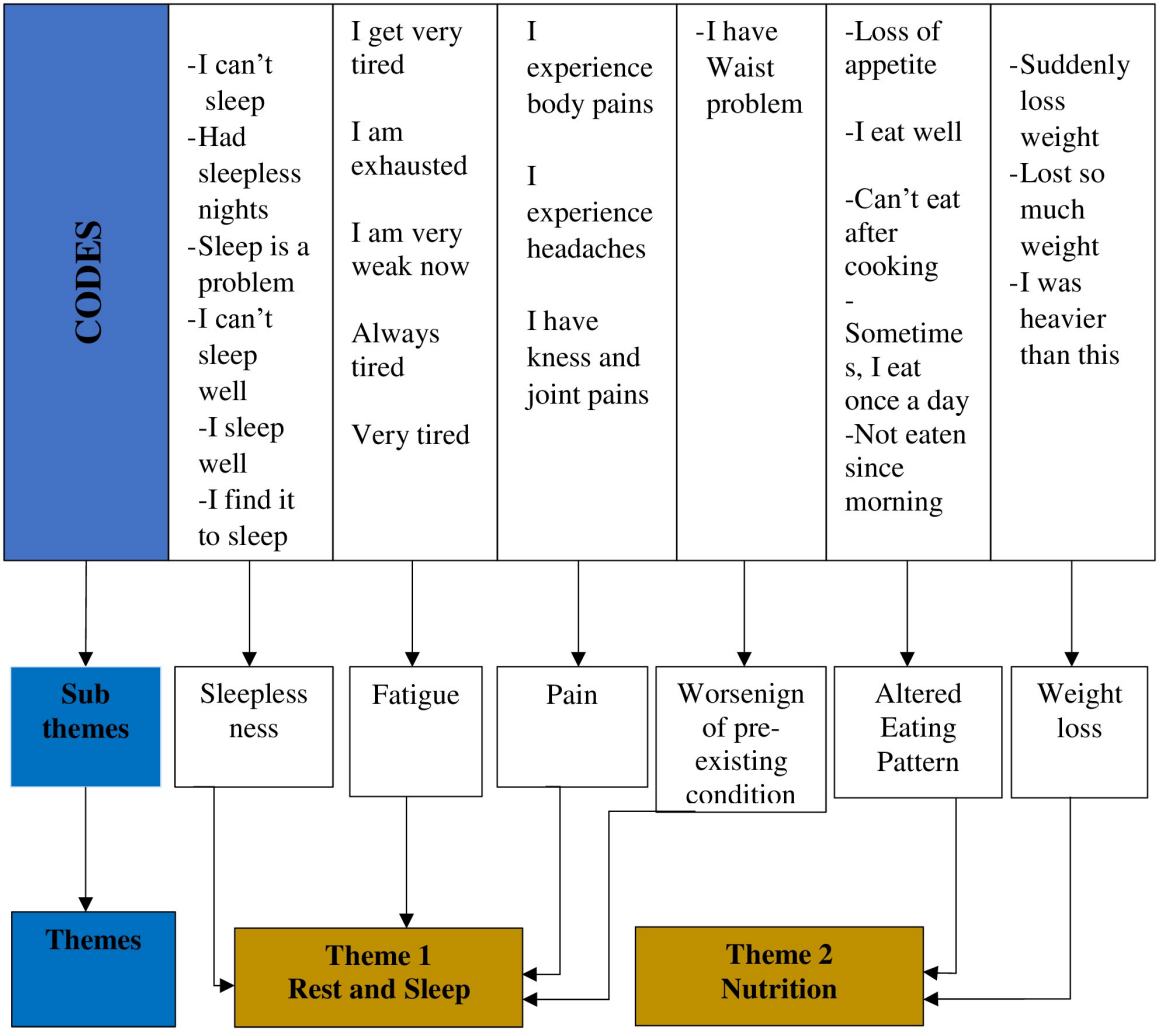
Coding tree for the physical impact of caregiving.

Each underlying meaning was coded in one category, reflecting a detailed description. ***Step four involved the grouping of acquired meanings together into thematic clusters***. From this process, central themes of “rest and sleep” and “nutrition” emerged with six (6) sub-themes. ***Step five followed with all sub-themes defined in a detailed description*** under the two main themes, “rest and sleep” and “nutrition”. After that, the researchers reviewed the findings in terms of richness and completeness to provide sufficient description and confirm that the detailed description reflects the experiences of the family caregivers of patients with prostate cancer. ***Step six involved returning to the participant for validation of the detailed description***. Participants’ views on the study results were obtained directly via phone calls. This step was done by the lead researcher as approval was taken earlier from the participants in advance during the first interview. Eventually, all participants showed satisfaction with these results, reflecting their feelings and experiences. ***The final step involved incorporating any new or pertinent data obtained during verification into the definitive study***. However, no further information emerged. Fig 1 below illustrates the coding tree of the themes and sub-themes that emerged from the analysis.

### Ensuring trustworthiness of the data

The study’s trustworthiness was achieved through four main criteria: credibility, transferability, dependability, and confirmability [34]. Bracketing was also used to validate the study’s trustworthiness further, as proposed by Husserl [35]. Data analysis was done using Colaizzi’s approach. This approach is the only phenomenological analysis that calls for the validation of results by returning to study participants, according to Polit and Beck [36]. Based on Lincoln and Guba’s [34] proposal, rigour was achieved through member checking with participants and other experienced researchers with different professional backgrounds. Transferability of our findings was ensured by providing a detailed description of our study background, methods, and conclusions and using a purposive sampling method to choose our participants.

On the other hand, dependability was strengthened by ensuring rich data grounding our analysis. Audit trails, including data collection and analysis processes such as audio files, transcripts, and field notes, were detailed and regularly consulted during data analysis and interpretation. Bracketing in the study was ensured right from the onset of the study, where the thoughts, feelings, and perceptions of the researcher(s) were ‘suspended’ and not allowed to bias the views and experiences of caregivers. Also, during data collection, the interview was guided by a scheduled prepared interview guide rather than dictated by it. In addition, the researcher probed freely on exciting areas of interest/concern that came up. Again, knowledge of the researcher being a nurse (which the participants were aware of) made it difficult for some participants to express their feelings/opinions frankly. Thus, the researcher ensured bracketing on both sides (researcher and participants), as suggested by Caelli [37]. In providing bracketing, the researcher had to “suspend” her opinions and experiences to view the phenomenon anew as it was experienced.

### Ethical considerations

Ethical approval was sought from the Institutional Review Board (IRB) of the University of Cape Coast (UCC1RB/CHAS/2018/24) and the Cape Coast Teaching Hospital (CCTH/RDS/2019/41). Copies of the ethical approval letter and an introductory letter from the School of Nursing and Midwifery, UCC, were sent to seek permission from the Cape Coast Teaching Hospital. All study participants signed/thumb printed an informed consent. Participants were assured of their anonymity, confidentiality, and privacy and were made to understand that participation in the study is strictly voluntary. Therefore, they can opt-out of the study at any time. The anonymity of participants was ensured by assigning pseudonyms to the participant during the recruitment. Pseudonyms were used when the participants were quoted in the findings section. Privacy was ensured during the interview, and soft copy files were saved up in a word document on a hard disc/email. Hard copies of the document were labelled with pseudonyms and kept in a file separately. Informed consent forms were saved in a locked file cabinet at the School of Nursing and Midwifery.

## Results

### Demographic characteristics of the participants of the study

The participant demographics suggest that most caregivers are females constituting about 90% of the study participants and largely the patients’ spouses. Nearly half of them have tertiary education. Many of them are either traders or are into farming as an occupation. Furthermore, the age of the caregiver’s averages between 40–60 years. In terms of religion, all the participants were Christians (see Table 1).

**Table 1:** Demographic characteristics of the participants of the study.

Pseudonyms	Age	Sex	Marital status	Religion	Educational level	Relationship with patient	Duration of Care	Occupation
Love	38 yrs	Female	Married	Christian	JHS (Junior High Sch)	Father	2 years	Seamstress
Peace	36 yrs	Female	Not Married	Christian	Tertiary	Father	1 year	Secretary
Grace	29 yrs	Female	Married	Christian	Tertiary	Father	1 year	Nurse (Awaiting postings)
Joy	58 yrs	Female	Married	Christian	Primary	Husband	1 year	Trader
Mercy	67 yrs	Female	Married	Christian	Tertiary	Husband	2 year	Retired
Forgive	62 yrs	Female	Married	Christian	Tertiary	Husband	1 year	Retired
Favour	41 yrs	Female	Married	Christian	SHS (Senior High Sch)	Father	1 year	Trader
Humble	62 yrs	Female	Married	Christian	Illiterate	Husband	1 year	Farmer
Passion	60 yrs	Female	Married	Christian	Illiterate	Husband	1 year	Farmer
Kindness	64 yrs	Female	Married	Christian	Tertiary	Husband	2 years	Trader
Hope	27 yrs	Male	Not Married	Christian	Tertiary	Father	6 months	Teacher
Faith	40 yrs	Female	Married	Christian	Illiterate	Father	1 year	Trader

### Physical impact of caregiving on the family caregiver

**Fig 2:**
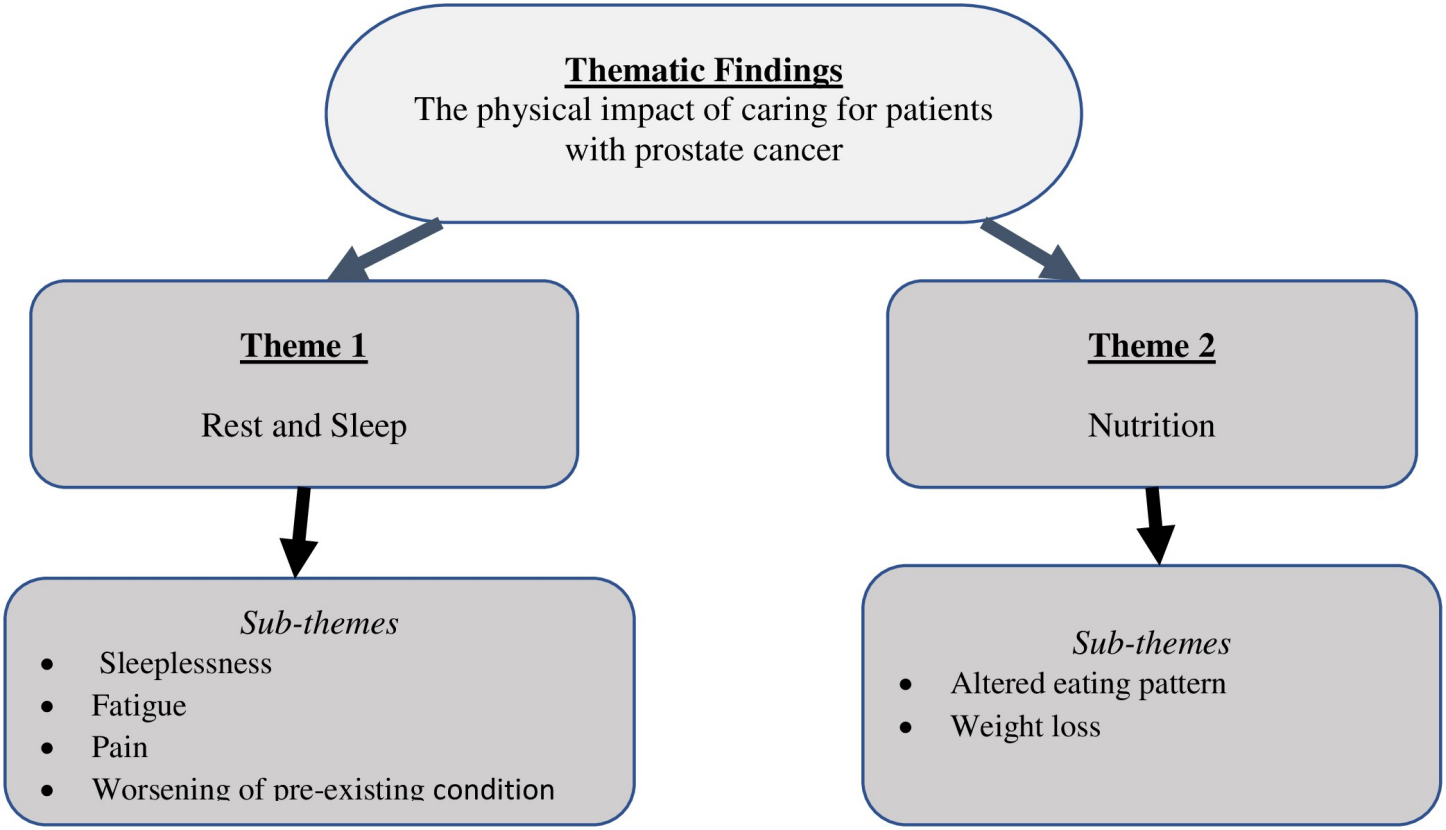
Themes and sub-themes.

The family caregivers shared their experience of the physical impact of their role in taking care of prostate cancer patients. They mentioned that caring for a relation with prostate cancer was a challenging task that resulted in much physical exertion. Especially when it involves constant washing/cleaning to do away with the stinging smell from leaking of urine due to urine urgency, lifting and changing patients’ positions, checking on patients from time to time, especially at night, accompanying the patient to the hospital, and managing the patient medications. However, the degree of impact depended on the severity of the condition and whether or not the patient was bedridden. Most of the participants stated that they suffered physical problems such as sleeplessness, altered eating patterns, pain, weight loss, fatigue, and worsening pre-existing conditions (see Fig 2).

### Theme 1: Rest and sleep

Ensuring adequate rest and sleep is especially important in the family caregiver’s life as it reduces stress, lowers the risk of heart diseases, and promotes energy regain. However, it is frequently overlooked due to the laborious nature of the caring role. Sub-themes that emerged were sleeplessness, fatigue, pain, and worsening pre-existing conditions.

#### Sleeplessness

Sleeplessness was a significant concern reported by most of the participants. Participants stated that sleeplessness resulted from staying awake to constantly check on the patient if they needed something or assistance. Others could not sleep due to persistent worry about the outcome of the patient’s condition and how to overcome the financial burden.

Participants could not sleep because the patient may need assistance in one way or the other.

*“Since this whole condition started*, *he could not sleep*, *so as a wife*, *I also find it difficult to sleep because if he is in pain or needs something and I’m fast asleep*, *I don’t know what will happen*. *Apart from those things he used to do on his own*, *he is no longer able to do them without assistance*, *so I must stay up and help”*.

***Mercy***

*“At night*, *I always have my mind on him*, *with the little noise from his room*, *I wake up to find out if everything is all right*. *Thus*, *I cannot sleep well”*.

***Grace***

Participants had sleepless nights because they were anxious and the patient was in severe pain.

*“Because of the situation*, *I can’t also sleep as the pain is too much for him to bear and also the pain makes him grind with his teeth which you could hear so loudly”*.

***Forgive***

Participants could not sleep due to the anxiety and persistent worrying about the patient’s health and how to overcome financial burden.

*“Sleep is a thing of the past*; *at the moment*, *what I do best is a worry*, *and worrying deprives you of your sleep*. *When I’m constantly worrying about his health*, *how to raise money for the medical bills*, *buy medications and pay for laboratory investigations*, *how can I sleep*? *Currently*, *the life we are living*, *hmmm… it is only God”*.

***Humble***

The participant does not live with the patient but goes home to her immediate family after rendering the care needed for the day. However, she reported having a sleepless night despite exhaustion, hence taking sedatives to enable her to sleep.

*“I always find myself exhausted*. *At night I find it difficult to sleep*, *so to have some good sleep and regain my strength for the more difficult task the next day*, *I sometimes take sleeping pills*. *I usually get them from the pharmacy”*.

***Favour***

#### Fatigue

Fatigue within this context is a subjective feeling existing at one point in time on a continuum from wariness to complete exhaustion resulting from physical, emotional and mental activities. Most of the participants expressed a feeling of severe fatigue due to physical activity. The degree of exhaustion depended on whether the caregiver lived with the patient in the same house or if they were the sole caregiver of the patient.

A Participant reported extreme exhaustion due to caring for her dad (patient) and her little girl.

*“I find myself working throughout the day*; *you know how urine is*; *if I don’t constantly wash the clothes and clean the room*, *no one can even sit there*. *Therefore*, *I’m always cleaning urine*, *especially with my little girl around crawling*, *touching*, *and inserting things into her mouth*. *Hmm*, *it is not easy for me at all*. *My day is full of activities*. *Activity after activity (from morning till evening) makes me very tired*. *Caring for little children is hectic and then adding a sick person to it becomes even worse…*.*”*.

***Grace***

The participant is the sole caregiver of a patient who is bedridden and dependent on her to meet personal care needs

*“He is also frail now*; *he can’t do anything for himself*. *Thus*, *if I am working on him*, *he cannot even help me*; *I will have to do everything myself*; *it is not easy*. *I am exhausted by the time I’m done coupled with other house chores”*.

***Joy***

Participants also shared their experiences with increased exhaustion due to the laborious nature of the caring role. The following were some of their responses:

*I am always tired*. *For instance*, *today*, *I had to wake up at 4 am and prepare everything for the children to take care of him*, *then rush down to the hospital to get his card and things ready and join the queue to see the doctor*. *Therefore*, *I had to take the lead to secure the place while he joined later*. *By the time we get home*, *hmm”*.

***Mercy***

#### Pain

The pain was a category that emerged under the physical impact. It is an unpleasant sensory experience associated with actual or potential damage. In this study, participants shared their experience of pain in four different ways, namely, general body pains, waist pain, headache, and worsening of a pre-existing condition. According to the participants, the experience of pain resulted from providing physical support, which includes assisting patients with daily activity, managing patients’ diseases, and medical appointments. Others also took ill due to the nature of the role they play.

*“Madam*, *hmm*, *I have knee and waist pain as we speak now*. *Sometimes I even want to give up*. *Because I am already tired of everything*. *My son used to bring him for reviews*, *but I had to come today because he is a teacher and he needs to go to work*. *Today*, *as I was preparing to bring him for the review*, *I had to wake up very early*, *prepare warm water to clean him*, *and dress him up*. *Look*, *I have severe waist pain*; *I am even sick*. *It is not easy”*.

***Joy***

#### Worsening of a pre-existing physical condition

Studies have shown that the caring roles were associated with developing chronic conditions and worsening pre-existing physical conditions. In this study, a participant stated that she had a waist problem that had become worse due to the demands of her caring role.

*“Caring for my husband is not easy*; *I have a waist problem*, *and I find myself always very tired*. *The waist problem was there but was not as serious as it has become now*, *all because of the role I currently play”*.

***Forgive***

### Theme 2: Nutrition

Nutrition is a critical part of the health of the family caregiver. A significant theme emerged with the sub-themes “altered eating pattern” and “weight loss”.

#### Altered eating pattern

The family caregivers shared their experiences on how the caring role has affected their eating habits. Most caregivers reported eating once daily or not eating at all except fluids because they had no appetite, which led to weight loss in some cases. This resulted from the increased workload making it impossible for them to make time for food.

Some of the participants indicated that because of the heavy workload in caring for the family, they had little time for themselves and thus affecting their appetite and eating habits.

*“My eating pattern*, *hmmm*, *I need to make sure my dad is served*, *and my girl too is served*; *by the time I’m done*, *I would have even lost appetite*. *What happens is that*, *after preparing breakfast*, *I may decide*, *oh*, *let me wash these few things*, *then before I know it*, *lunchtime*, *I get their food ready*, *and something else comes up again*, *so I sometimes I end up eating once and mostly in the evening”*.

***Grace***

Another participant has this to say

*“Sometimes I eat once a day because there is too much to be done*, *and by the time I realise it is already time for dinner”*.

***Favour***

Some participants reported they could not eat due to the persistent worrying about the health of their loved ones.

“*Yes*, *I haven’t eaten anything since morning*, *but I want him to eat something and recover quickly*. *I’m not worried about me but more worried about him*. *Because I believe that if he is okay*, *I will also be fine”*.

***Faith***

#### Weight loss

Participants reported weight loss due to poor nutritional intake. This was attributed to their inability to make time for food and persistent worrying about the patient’s condition.

At the same time, others attributed the weight loss to constantly worrying about the patient.

Participants shared their views.

*“Sometimes it is difficult to eat because I cannot make time*, *but I try to make sure I take in something*, *since I don’t want to lose weight as my colleagues at work were already complaining that I have lost too much weight”*.

***Peace***

*“Madam*, *I am scared and always worried because of the condition*; *I was heavier than this*; *look*, *I have lost so much weight since this condition started*. *I have changed”*.

***Joy***

## Discussion

This study sought to explore the experiences of family caregivers of prostate cancer patients regarding the physical impacts it has on them. The findings show that the physical impact of caregiving is closely associated with the physical tasks that caregivers are expected to perform. As the condition advances for the patient with prostate cancer, so does the caregiver experience increased physical demands. The caregivers assume additional roles performed by the prostate cancer patient/change in roles as their disease progresses. Family caregivers’ specific physical impacts were sleeplessness, fatigue, and altered eating patterns. Others also reported having experienced pain (headache, waist pain, body pain), worsening of pre-existing pain and weight loss.

### Sleeplessness

Lack of sleep was a major concern for most family caregivers. Sleeplessness results from persistent worrying about the outcome of the condition and financial burden. Also, they have to check on the patient constantly. Others reported taking sedatives to induce sleep. This finding is consistent with Ofori’s [38] findings that showed that participants had sleepless nights and others had to take sedatives to help sleep. These findings are further echoed in the works of Aziato and Adejumo [39], Carter [15] and Glajchen [14]. Therefore, it is well established in the literature that sleep deprivation is a significant issue experienced by family caregivers of cancer patients. It is viewed as problematic as it causes daytime fatigue and depression for a long time. Thus, it affects the family caregiver and the quality of care provided to the patient [14, 40–42]. However, sleep deprivation or insomnia was not a concern to other family caregivers. This may reflect the relationship between the patient and caregiver because the patient may not want to discuss their problems with the caregiver and suffer in silence since they do not want to bother the caregiver [43].

### Fatigue

As a result of the increased demand for caregivers of prostate cancer patients, fatigue among family caregivers is a growing health concern. Family caregiver fatigue is associated with poorer quality of care for the patient. The current study demonstrated that fatigue is a common symptom among family caregivers of patients with prostate cancer due to physical activities’ burden and strain. These findings are similar to Ofori’s [29] study, which reported that the daily assistance provided to patients with prostate cancer resulted in spousal fatigue. Fletcher et al. [41] and Yao et al. [13] mirrored the findings of this study by asserting that family caregivers of patients with prostate cancer experience fatigue due to the difficulties involved in assisting patients with activities of daily living.

Furthermore, the findings of this study showed that participants who were employed experienced more fatigue as a result of combining their job with the caregiving role. Campbell et al. [44], in the study of Prostate cancer in African Americans: relationship of patient and partner self-efficacy to quality of life, revealed that caregivers considered fatigue as an acceptable emotional response to taking care of a spouse/partner who has been diagnosed with prostate cancer. Thus, they considered fatigue as a normal response as far as they can care for their spouse/partners effectively. These findings, however, contradict the results of this current study. Furthermore, although not evident in this study, Ofori [38] revealed that the older the participants, the more likely they complain of fatigue.

### Pain

Participants commonly reported pain. The experience of pain, according to participants, is as a result of the physical demand to help patients with daily activities (such as lifting, changing position, turning patients in bed), managing patients’ diseases and medical appointments. These findings are consistent with the studies of Given et al. [45] and Ofori [38] who reported that caregivers experience headaches and severe body aches due to the caregiving role. Several other studies have documented that family caregivers of patients with cancer experience problems such as headaches and muscle pain [46–48]. Therefore, it is imperative that as nurses, these family caregivers are provided with the skills and training needed to carry out the role to reduce this detrimental effects on their physical health. For example, training on moving and handling techniques may reduce injuries and promote caregiver health.

### Worsening of a pre-existing condition

Participants reported worsening pre-existing physical health such as waist problems in the process of caring. This finding is supported by Ofori [38], where participants presented an increase in their blood pressure with associated pre-existing conditions such as diabetes and asthma. These findings are also similar to what Northouse et al. [49] reported in their study.

### Altered eating pattern

The altered eating pattern was another key concern among family caregivers. This was primarily attributed to the increased workload making it impossible for them to make time for food. Similar findings were reported by Ofori [38]; however, in her study, participants had to engage in fasting, which prevented them from eating and as a form of prayer. However, some participants reported a regular eating pattern; this suggests that there are situations where the caregiver’s eating pattern is not affected despite the stress involved in caregiving. Furthermore, reduced appetite is reported by other studies [50, 51]; in particular, family caregivers who have to feed their loved ones daily develop a poor appetite for food.

### Weight loss

Some participants reported weight loss due to poor nutritional intake, which was attributed to their inability to make time for food and persistently worrying about the patient’s condition. This finding is consistent with a study by Sercekus et al. [52], who reported that participants experienced a loss of appetite and weight loss due to the stress of caregiving. This finding was further validated by Hopkinson [53], who revealed in a study among family caregivers of patients with advanced cancer and eating problems that these family caregivers lose weight due to neglecting their nutritional needs.

## Strength and limitations of the study

This study had some limitations which need to be highlighted to allow for contextual interpretation of the study findings. First, the study was limited to only the caregivers, without the perspectives of the patients themselves and the health professionals. Similarly, the study purposively selected twelve family caregivers, and since this is a qualitative study, the evidence cannot be generalised. Despite these limitations, the qualitative approach was the methodology of choice, giving the focus of this study to emphasise the lived experiences of caregivers of patients with Prostate cancer in this setting.

### Policy implications

Cancer treatment in Ghana is costly. Many cannot afford to keep their loved ones in hospitals, thus requiring policies to support family caregivers who cannot combine or give up their jobs to care for the patient. This support may be given by enacting policies that allow the cost/treatment for prostate cancer to be absorbed totally by the Ghana National Health Insurance Scheme. Also, paid leave policies can be implemented to increase financial support for workers providing essential care for family members with prostate cancer. Additionally, developing and implementing educational training programmes will improve the quality of life for family caregivers of patients with prostate cancer.

## Conclusions

The study revealed that family caregivers of patients treated for prostate cancer experience physical consequences due to the caregiving role, impacting their physical health. It is of great importance, especially for nurses, to come up with measures to minimise these adverse physical effects on the family caregivers through formal education programmes/ training on how to care for these patients at home. We hope that this study will further stimulate additional research to bridge the gap in our knowledge on the physical impact the caregiving role has on the family caregiver and provide the necessary support needed.

## Supporting information

S1 Appendix Coreq checklist. (DOCX)

S2 Appendix Data collection instrument. (DOCX)

S3 Appendix Transcripts. (DOCX)

S1 File (DOCX)
