# Supplementary material for: “I always find myself very tired and exhausted”: The physical impact of caring; a descriptive phenomenological study of the experiences of prostate cancer caregivers in Cape Coast, Ghana
Source: PLoS One. 2022 Jul 26;17(7):e0268627. doi: 10.1371/journal.pone.0268627 (PMC9321373; doi:10.1371/journal.pone.0268627)
Supplement: S1 Appendix — (DOCX) [file pone.0268627.s001.docx]

**S1 APPENDIX**

## Manuscript: “*I always find myself very tired and exhausted”*: The physical impact of caring; a descriptive phenomenological study of the experiences of prostate cancer caregivers in Cape Coast

**Consolidated criteria for reporting qualitative studies (COREQ): a 32-item checklist**

Developed from:

Tong A, Sainsbury P, Craig J. Consolidated criteria for reporting qualitative research (COREQ): a 32-item checklist for interviews and focus groups. *International Journal for Quality in Health Care*. 2007. Volume 19, Number 6: pp. 349 – 357

| No. Item | Guide questions/description | Remarks | Reported on Page # |
| --- | --- | --- | --- |
| Domain 1: Research team and reﬂexivity |  |  |  |
| *Personal Characteristics* |  |  |  |
| 1. Interviewer/facilitator | Which author/s conducted the interview or focus group? | The interviews were conducted by BO | Page 6 |
| 2. Credentials | What were the researcher’s credentials? E.g. PhD, MD | The researchers’ credentials are as follows:  BO: RN, BSc, Mn  JPN: RN, PhD  EAA: RN, PhD  AS: BA, PGDE, MPhil | Page 1and 8 |
| 3. Occupation | What was their occupation at the time of the study? | The researchers’ occupations are as follows:  BO: Nurse  JPN: Lecturer/Academic  EAA: Lecturer/Academic  AS: Lecturer/Academic | Page 1and 8 |
| 4. Gender | Was the researcher male or female? | The researchers gender are as follows:  BO: female  JPN: Male  EAA: Female  AS: Male | Page 1 and 8 |
| 5. Experience and training | What experience or training did the researcher have? | BO has a MN degree/attended a workshop on “how to conduct qualitative research”. JPN, EAA and AS are experienced researchers in qualitative studies and have collectively published numerous qualitative research articles. | Page 1 and 6 |
| *Relationship with participants* |  |  |  |
| 6. Relationship established | Was a relationship established prior to study commencement? | Yes but only for the purposes of this research. | Page 6 |
| 7. Participant knowledge of the interviewer | What did the participants know about the researcher? e.g. personal goals, reasons for doing the research | \| None of the participants knew the researchers. However, all participants knew that the interview was for research purposes only. \|  \| \| --- \| --- \| | Page 7 |
| 8. Interviewer characteristics | What characteristics were reported about the interviewer/facilitator? e.g. Bias, assumptions, reasons and interests in the research topic | The characteristics of the researcher were that she is a practicing nurse from a different hospital outside of the region where the study was conductored. | Page 10 |
| Domain 2: study design |  |  |  |
| *Theoretical framework* |  |  |  |
| 9. Methodological orientation and Theory | What methodological orientation was stated to underpin the study? e.g. grounded theory, discourse analysis, ethnography, phenomenology, content analysis | descriptive phenomenological approach was applied. | Page 5 |
| *Participant selection* |  |  |  |
| 10. Sampling | How were the participants selected? e.g. purposive, convenience, consecutive, snowball | They were selected purposively | Page 5 |
| 11. Method of approach | How were participants approached? e.g. face-to-face, telephone, mail, email | They were approached face to face | Page 7 |
| 12. Sample size | How many participants were in the study? | There were 12 participants in all | Page 7 and 8 |
| 13. Non-participation | How many people refused to participate or dropped out? Reasons? | None | Page 6 |
| *Setting* |  |  |  |
| 14. The setting of data collection | Where was the data collected? e.g. home, clinic, workplace | Data was collected at homes of participants, within the hospital premises, at the hospital snack bar/eatery, and outside some of the wards | Page 7  . |
| 15. Presence of non-participants | Was anyone else present besides the participants and researchers? | No-one else was present besides the participants and the researcher | Page 7 |
| 16. Description of sample | What are the important characteristics of the sample? e.g. demographic data, date | The important characteristics of the samples were their age, gender/sex, marital status, relationship to patient, duration of care and occupation. | Page 11 |
| *Data collection* |  |  |  |
| 17. Interview guide | Were questions, prompts, guides provided by the authors? Was it pilot-tested? | A guide was prepared (see S2Appendix), and pilot tested with 2 participants. | Page 6 |
| 18. Repeat interviews | Was repeat interviews carried out? If yes, how many? | No repeat interviews were carried out. | N/A |
| 19. Audio/visual recording | Did the researcher use an audio or visual recording to collect the data? | Interviews were audio recorded. | Page 7 |
| 20. Fieldnotes | Were ﬁeld notes made during and/or after the interview or focus group? | Field notes were made by BO after every interview. These field notes were used to assist in the analysis of the transcribed audio recordings. | Page 7 |
| 21. Duration | What was the duration of the interviews or focus group? | The duration of the in-depth interviews ranged from 40 to 50 minutes. | Page 8 |
| 22. Data saturation | Was the data saturation discussed? | Data saturation was discussed in the methodology section. | Page 7 and 8 |
| 23. Transcripts returned | Were transcripts returned to participants for comment and/or correction? | Participants were called on phone for comment or correction. | Page 8 |
| Domain 3: analysis and ﬁndings |  |  |  |
| *Data analysis* |  |  |  |
| 24. Number of data coders | How many data coders coded the data? | Three authors coded the data (BO, JPN and EA) | Page 8 |
| 25. Description of the coding tree | Did authors provide a description of the coding tree? | Description of the coding tree has been provided in the methods. Analysis was undertaken concurrently with data collection to check for data saturation. | Page 9 |
| 26. Derivation of themes | Were themes identiﬁed in advance or derived from the data? | The themes were derived from the data during analysis. | Page 12 |
| 27. Software | What software, if applicable, was used to manage the data? | No software was used | N/A |
| 28. Participant checking | Did participants provide feedback on the ﬁndings? | Yes, participants provided feedback on the findings through phone calls. | Page 8 |
| *Reporting* |  |  |  |
| 29. Quotations presented | Were participant quotations presented to illustrate the themes/ﬁndings? Was each quotation identiﬁed? e.g. participant number | Yes, verbatim quotations were presented to illustrate the themes/ﬁndings. | Page 13 to16 |
| 30. Data and ﬁndings consistent | Was there consistency between the data presented and the ﬁndings? | Yes, there was consistency between the data presented and the findings. | Page 12 to 16 |
| 31. Clarity of major themes | Were major themes clearly presented in the ﬁndings? | Yes, major themes were clearly presented in the findings. | Yes. they were.  Page 12-16 |
| 32. Clarity of minor themes | Is there a description of diverse cases or discussion of minor themes? | Yes, there was a Discussion of major and minor themes. | Page 17-20 |
