# Supplementary material for: “I always find myself very tired and exhausted”: The physical impact of caring; a descriptive phenomenological study of the experiences of prostate cancer caregivers in Cape Coast, Ghana
Source: PLoS One. 2022 Jul 26;17(7):e0268627. doi: 10.1371/journal.pone.0268627 (PMC9321373; doi:10.1371/journal.pone.0268627)
Supplement: S2 Appendix — (DOCX) [file pone.0268627.s002.docx]

**S2 APPENDIX**

**DATA COLLECTION INSTRUMENT**

**Interview Guide**

**SECTION A**

1. **Demographic Information**

Identification (ID) Code ………………….

Age

Sex

Nationality

Highest educational level

Occupation

Religion

What is your relationship with the patient?

**SECTION B**

1. **What are the experiences of caregivers caring for people with prostate cancer?**
2. How long has your relationship been living with the condition?
3. How long have you been providing care for the patient?
4. Please kindly tell me about your relation’s condition
5. What exactly do you usually do for him daily?
6. How do you think your caring role has affected you physically?

Probes:

Does your caring role affect your eating pattern?

How does your role affect your sleeping pattern?

Do you usually get tired?

**SECTION C**

1. **Please, is there any other thing you would like me to know?**
