# Supplementary material for: “I always find myself very tired and exhausted”: The physical impact of caring; a descriptive phenomenological study of the experiences of prostate cancer caregivers in Cape Coast, Ghana
Source: PLoS One. 2022 Jul 26;17(7):e0268627. doi: 10.1371/journal.pone.0268627 (PMC9321373; doi:10.1371/journal.pone.0268627)
Supplement: S3 Appendix — (DOCX) [file pone.0268627.s003.docx]

**S3 APPENDIX: TRANSCRIPTS**

**NB: ALL NAMES ARE PSEUDO NAMES**

Participant: Love

Love was met at the XX on the 2/04/2019. She had come to the clinic with her brother and their dad who is a prostate ca patient. It was the review date to see the urologist for an earlier scheduled orchidectomy to be performed tomorrow 5/04/19. I had the opportunity to meet the brother and their dad a the consulting room, where I took the chance to explain to them what my study was all about and its importance within our context. It was then that the brother told me about Love and the fact that she was the sole informal caregiver of their dad.

I eventually met Love outside the consulting room, after the patient had been seen by the urologist and admitted to the ward against the surgery scheduled for tomorrow. I again took the opportunity to explain to her what the research was all about. She was happy to be part of the study. We then scheduled a convenient time and place where the interview can be conducted.

At 4pm we met at the snack joint within the hospital premises for the interview. Consent form was signed and the interview commenced after assuring the caregiver of annonymity.

Interview lated for 37 minutes/21 secs

Me: Tell me alittle bit about yourself, interms of your level of education and your age please

Love: I completed junior secondary school. Me: Oh okk…Love: I am XX years.

Me: Who is the patient to you please?

Love: He is my dad

Me: Please are you the sole caregiver of your dad? Love: Yes pls. Are you the one who takes care of him solely at home? Love: Yes pls. Me: So you mean you take care of him everyday? Love: Yes pls

Me: Please what do you do for a living?

Love: I am a seamstress. Me: wow…So are you able to go to work?

Love: Yes I am able to work but since he took ill, most of my clients have come for their cloths because I’m not able to make time to saw for them. Me: hmm. Love: Some of my clients understand me when I tell them about the situation but others don’t so I have to return their cloths back to them. Me: You mean you give it back for them to go and saw them somewhere else because you wouldn’t make time to saw? Love: Yes, I have even cut some of them but because of the current role I play, im not able to make time to saw. Me: eeiii, then you must be in deep heat. Love: yes oo it is not an easy situation. Me: eeeiiiiii, this must be very serious

Me: Do you have children? Love: Yes. Me: so what about your husband? Yes. Me: Do you leave together with your immediate family?

Love: No, my husband and children are in Mankessim. Me: oh k, so how do you meet your family especially your husband?

Love: I go there once awhile, mostly on weekends

Me: So, who takes care of dad when you are away?

Love: No one takes care of him. And since I took up this role my marriage has been on the rocks. Me: eeeiii really? Love: Yes, because my siblings and I are about 7 in all. 2 males and 5 females but they have all left the caregiving role on me just because my home and that of my dad’s is closer. Me: How are you nearer to him. Love: I live with my family at Mankessim and my dad is at Beseasi, few minutes’ drive from mankessim where I live. Mostly going there from my place will cost about 1 cedi 50 pesewas. I cant go and return everyday hence, needed to stay with my dad. My husband is not happy about this decision at all and also with the fact that my other siblings have left everything on me. He believes that, the females should come together and try to care for our dad in turns. They are also married and are with their husbands. Me: hmm hu. So that the same way I leave him, they can also leave their husbands for a while. In fact, this is a serious issue between the two of us now. Some time when I call him he will not even answer my calls. If he does then it’s all quarrels. Every trivial thing becomes a misunderstanding between us. Me: wow. Love: I can’t also leave my dad and go and sit home doing nothing. He took care of me, so, now that he needs me I can’t turn my back on him. The bible even tells us to honour our father and mother. So, will continue to do this and see what God has for me. My brother, the one you met at the doctors room, he only comes around when we are comig for review. He does not live with my dad and I. Anytime he comes with us to the hospital, he goes to sleep at Ankafo at a friend place. Me: You see what I mentioned earlier (Off record…) that it was necessary for the hospital to have a place for caregivers to lay their heads. Love: Yes oo. Even me.. I would have stayed if there was a place I could rest. Again, if there is some thing to be done for the patient and you are not around it wont be done until you return yet there is no place here to stay whiles your relation is on admission.

Me: What about your mother?

Love: my mother is alive but not doing well, so she can’t take care of my dad.

Me: so, in this case you will have to go home this evening since dad has been admitted.

Love: Yes.

Me: Then, I guess you will get to go and spend some time with your husband and children before dad is discharged.

Love: No, I can’t because tomorrow morning I will have to return back to the hospital.

Me: Oh ok

Me: So, it means the children are with your husband now. Love: Yes. Me: How many are they?

Love: They are 3. Two boys and a girl. The eldest is a boy. He is 15 years old now.

Me: About the current problems between you and your husband, have you two tried to come together to find a common solution to it. You know that one of the most important things in marriage is communication. I believe that if you try to talk about it you both may find a solution that will favour you both.

Love: Hmm the truth is sometimes I go there after 2 weeks, even after a month. I know he is really not happy about this at all but his major problem is that, my other siblings should also leave their husbands and go and take up the role just as I leave him. It should be on shift basis and not to be left for just one person. I have tried to speak with my siblings but they are also not ready to do it. So, because of this he doesn’t understand anything I tell him. He is not ready to even listen.

Me: Tell me about your dad’s illness. How it all started.

Love: It all started two years ago in the month of August. My husband is a teacher, so we use to live in XX but we were later transferred to XX. During that time there was a festival, which we attended together. But my dad had a little injury whiles going about his normal daily activities as a farmer. The injury was from a stick, he even asked that I applied a perfume on the site so I did. Three days after the injury he started complaining of feeling cold. So, he went for tetanus injection. My sisters were around at the time, as they have also come for the festival. One of them told him to go to the hospital if the condition is not improving and he agreed. But unfortunately, the affected limb became very swollen the next day. So, I called my sister to inform her and my she asked that we take him to a clinic in Takoradi called XX clinic which we did. He was on admission for 3 weeks while treatment was carried out. We spent a lot of money. I may say we spent over 8,000 Ghana cedis. We then asked for a referral to a hospital within Cape coast in order to cut down on cost. We were then referred to a hospital in Assikuma where we continued the treatment. Finally, the leg was healed, he had stopped attending the clinic. Suddenly, on one faithful night my mum called to inform me that my dad can not pass urine. So, asked her that, what illness is that? That night we quickly took him to XX hospital where a rubber was passed to drain the urine and then we took him home. He started going for reviews at XX since that is the hospital he mostly attended. But suddenly he started complaining that, he the urine is not passing through the catheter any longer. So, he went back to Ajumako and this time he was referred to XX Hospital. When we got here, a rubber was put in his lower abdomen, and the urine was drained from there. We were also made to do some labs and later told that he will need a surgery to be done urgently. We were required to make a deposit of 1000, that same day so that the surgery can be done the following day but unfortunately for us we could not raise the amount so we had to go back and come after we raised the money. But before then we continued the review tell we were able to raise the money. By the grace of God, we have paid the money but the date scheduled for the surgery was cancelled after all preparations done and we had to send my dad home again. The reason for cancellation was not communicated to any of us yet he was discharged to be brought back a week later. Me: Hmmm…tossing patients up and down. Love: Now he has been admitted again, so we are hoping that this time the surgery will be done.

Me: So please tell me what you do for him on daily basis?

Love: In the morning I go check on him, to find out if he is already up. If he is still asleep, I go ahead with other things I need to do. “Nukr3 di3 aye d3” But if he is already up, I go on to boil water to clean him up and then assist him with brushing of his teeth. I then go ahead to find out what he wants to have for breakfast, then I prepare it for him. He is not able to do much for himself. He sits in the wheelchair all day, so cleaning him up is a very difficult task and especially when I have to assist him when he wants to visit the toilet. There is no toilet in the house hence, I must lift him unto a chamber pot, and then lift him back again unto the wheelchair with no one to assist. But on weekends I get a little assistance from a brother in the house. After which I have to empty the pot. Depending on the weather temperature, I may clean him with warm water or tepid water. At lunch I prepare what he requests for him. Mostly banku with light soup and then something light for supper. Me: eeeiii this won’t be an easy task at all, you are really doing well. God bless you.

Love: Yes, oo it is not easy. Sometimes I even fall sick and have to go to the pharmacy to get myself some medications. I normally have body pains and feel weak. In the mornings, waking up from bed is very difficult because I experience pains all over my body. He is very heavy as you see him there like that.

Me: Are you able to sleep? Or sometimes you go for some of these sleeping pills to help you sleep?

Love: oh no, I sleep in a different room from my dad. He sleeps next to my mum but my room is next door. Mostly, when he is in pain, he makes the hm..hm.hm sound so I quickly go in there to find out what the problem is. He will tell me that he is tired of sleeping for a long time in a particular position. In this case I have to try and do some turnings or sit him up in bed, the next minute he will call again for you to assist him assume another position Because of that I can’t sleep as my mind is mostly on him throughout the night.

Me: So, what about your eating habits. Are you able to eat well?

Love: Hmm sometimes immediately you try to put some food in your mouth, he will call you to assist in easing himself. Me. Oh ..wow. Love: yes, so what happens is that you end up not able to continue with your food. I have to then get some drink since I’ve suddenly lost appetite…Me: Laughing…

Me: So, what are your children saying? Are they not complaining?

Love: No oo, they call every day. I tell them grand paa is not well so I have to take care of him and they need to pray for him to get well. When he recovers, I will return home to be with them. Also, anytime I go around I make sure I spend some quality time with them. Even though they are not happy about the situation there is nothing they can do about it so they understand.

Me: Do you sometimes feel overwhelm with everything and the thought of giving up comes into mind?

Love: Yes, most of the times. Especially when he starts complaining about things. For instance, when he becomes difficult to please and everything isn’t good enough. It is very frustrating.

For instance, this morning when we got here, I had to get him millet porridge but I could not get any accompaniment, so I told him to go ahead and have it. I will get him his favourite waakye soon after they come around and he agreed. After some few minutes the waakye was in so I decided to get it for him but he refused. Not long after, my brother came and he told him to ask me to buy the waakye for him…(Laughing) Me:… laughing.

Me: So, when you feel like giving up on everything. What do u do? Do u seek counsel from somewhere, someone etc.

Love: Yes, I do. Sometimes my mum encourages me, people from church also comes around to talk to me especially when they come to offer communion to him, commending me for the job I’m doing. I also encourage myself sometimes. I believe that God will heal him soon.

Me: So, tell me how you felt when you were told that your dad could not pass urine. Before then you told me earlier that you do not know exactly what your dad’s condition was?

Love: In fact, I felt someway (Me nipadwa po yee me bribi). Because I had no idea that there is a condition that can make someone not to be able to urinate. As human as we are if you can’t urinate then it is a big problem. But when I realised that something can be done about it. I was relieved.

Me: Have you heard of cancer?

Love: We were told that there is cancer in his spinal cord but after the operation everything will be ok.

Me; So, when you heard that there is cancer in your dad’s spine cord, how did you feel?

Love: People say it is a condition that can not be cured so it is scary.

Me: hmmm oh ok

Me: Some people say it is infectious, have you heard something like that

Love: The other day Sani (a nurse at the urology clinic) explained to us that cancer is not an infectious condition. Me: oh ok that is good. Sani did very well then.

Me: what about your social life. Are you able to interact with your friends? Go out with friends etc

Love: yes, but it depends on the time, and also it has to be brief so that I can quickly come back home to take care of my dad. That is if I have to attend important functions. In terms of grooming I’m able to take care of my looks

Me: So, what about the financial responsibilities.

Love: We do contributions.

Me: what about loans

Love: no, we don’t take loans

Me: So, during your previous stay on the ward, did any of the nurses sit any of you down to explain to you what exactly was going on and what you need to do when you go home?

Love: No please

Me: are you sure? Or you were shy and so even though they didn’t, you also didn’t ask?

Love: oh no. I may say the opportunity didn’t come for me to do that. But when we were discharged, a gentle man in brown approached me to remind me of the wound dressing every three days.

Me: So how do you scheduled your duties in a way to be able to make personal time

Love: I plan my day, in so doing I, able to make time for myself

Me: Is there any thing you would love to discuss with me? Something we haven’t mentioned.

Love: One thing I’ve always wanted to asked. People say the man is old so doctors telling us that they need to do surgery are all lies. They just want to take our money

Me: oh, not at all. The surgery is actually one of the treatments for prostate cancer. When surgery is done to remove the cancerous gland, it helps to reduce the chances of the cancer spreading to other organs within the body thereby increasing survival rate. So, it is never true that the doctors and nurses want to take money from you.

Love: oh ok, thank you, now I understand

Me: Thank you too very much

Participant: Peace

Age: XX yrs

The patient was met at the consulting room of the urology clinic where they had come for review. Patient came in with his brother in a wheelchair. At the consulting room, I introduced myself and explained the study. I then went ahead to find out if the gentle who had wheeled the patient in to the consulting room was the caregiver. But I found out that the caregiver was at home but the brother of the patient was the one who usually brings him for review. I decided to take the number of the brother so that I can visit home where I can get the opportunity to meet the caregiver. A convienient date and time was scheduled.

On the 3/4/2019 at 6pm I found my self in the home (Somewhere called XXX near XXX) of the patient after informing them of my visit earlier on during the day.

The interview began with an explanation on the relevance of the study, followed by the signning of the consent form.

Observation made: The caregiver was strictly monitored for reasons I don’t know of. The patient and his brother wanted the interview to be conducted in their presence, meanwhile from the few interwiews I have done, It has come to my realization that, the caregivers are not able to express their feelings freely in the presence of the patients or another person other than the interviewer. So, I insisted to conduct the interview away from their sight but assured them that we were only going to discuss issues in line with the care she provided. Yet, the uncle stood close to the where the interview was conducted till we finished with everything. I was really surprised. It made eliciting information from the caregiver very difficult, as you could see that she was not very comfortable.

Interview lasted for 40minutes/ 50 secs

Me: Can you please tell me your age?

Peace: XX years

Me: please, what about your level of education

Peace: Tertiary

Me: what do you do for a living please?

Peace: I work with African Asante Tour, a tour operating campany. I work as the secretary of the company. We make the arrangements and bookings for the patients

Me: So, what is your relationship with the patient?

Peace: He is my dad

Me: Please tell me what you know about the condition?

Peace: I know it is prostate enlargement and cancer

Me: Oh ok

Me: Tell me more please, how it all stated?

Peace: Hmmm

Me: When, how long has it been, when it all started

Peace: Hmm… I think he started complaining of the waist pain

Me: when was this,

Peace: I think early part of last year. Me: so its over a year now? Peace: yes, I think 17^th^ june, fathers day, he started complaing of waist pain the night after fathers day. He said he couldn’t sleep, it was more severe than the previous pains he has been having. So that night we had to go to the hospital.

Me: Where? Which hospital

Peace: The one at Tantre ehh…Me: XX? Peace: yes XX

Peace: He said the leg was paining him, so, they had to carry him home from the hospital. I wasn’t around but I came because of the illness. At the hospital he called me onphone to tell me everything that had happened. So, when he came home, the next day, it was a tuesday, he called me again around 5:30am and said when they went to the hospital things don’t look good at all. Things are bad and even now he cant even walk or lift his legs. The legs are powerless. So when I came home we went to another hospital, a herbal hospital.

Me: oh ok, what happened there too?

Peace: There, we went through the proceedure. They checked him and all that. Then he was given medicines, all natural medicines.

Me: During all this time, did he not have any problem with urination?

Peace: I think when they did the checking, they asked him some questions about the urination and he said that’s true he had problem with the urination and that was connected with the waist pain. So after that he was given some drugs and all that to take home. On that Tuesday when we got home he was still complaining that the urine was not coming and he was also having pain in his abdomine. So, one of my cousins suggested that we go to the Reginal hospital. Me: ooh ok.

Peace: so, when we went there, they fixed the catheter and immediately the urine started flowing. He was then admitted for about three weeks or so. Me: At the reginal hospital? Peace: yes, and then later he was discharged home. So, when he came, then I have to be around because I can not go back. Me: Ahhhh ok

Me: so, it was during the admission that they did the surgery? Peace: yes. Me: then after the surgery he was dicharged home? Peace: yes. The catheter was still there after discharge, that is from june to january when it was removed.

Me: So, what came to mind when you heard that he had cancer? You know a lot of people have different perception of what it is. Peace: hmmmm.

Me: Imagine you are there and suddenly you get a call that dad is not well. Your heart may skip a beat. So just tell me your experience, how you felt.

Peace: Hmm, I didn’t feel good anyway.Me: Huh huh. Peace: I didn’t feel good, my sisters are in Accra I’m the only one here. Me: how many are you? Peace: We are three, all girls. When he was admitted they came, but I was the one doing all the rounds everyday. I don’t return home when I go to the hospital. But if my uncle comes around then I go home to get some few things and return back to the hospital. Me: huh huh. Peace: it was not easy at all.

Me : ok, I want you to describe the experience, that’s what im interested in, because with that, you are the best person who can give me a detailed decription of how you felt and what happened.

Peace: It was not a good experience, I was always thinking about him. Even my colleagues at work, they even told me that Ive suddenly lost weight and that its just the beginning so I should take heart, everything will be fine. God is in control. My collegues from work were encouraging me. It took some time though, because when he was discharged my sisters also left so I was taking care of him alone. Some times my uncle is also around to help but not all the time because he goes to Elmina to do some small job. Along the line we employed someone to help when we are both not around.

Me: the person you employed, is he/she a nurse?

Peace: oh no. The person was just here to help him if he needs something and not to take care of him. I provide everything before I go to work.

Me: Earlier on you mentioned encouragement. So during the difficult times did you ever seek the assistance of a counsellor? I hope you know that the hospital has counsellors.

Peace: No oo, I never sought the services of a counsellor

Me: Did you ever seek support from church, friends etc

Peace: Yes, especially financial support from one or two friends because the money was just going like that. And I needed money to buy one or two things, sometimes medicine. Me: what about church? Peace: oh no. Me: Where do you fellowship please?

Peace: “Abandant life” They were aware of it anyway. Me: hmmm. Peace: But we sought seek support from them. Me: And they never attempted to assist in anyway? Peace: They do come around to pray for him and give communion. Me: what about you? Tell me more about that.

Peace: They come to visit, I think from the beginning stages they were coming every Sunday to encourage him and pray. Me: so what about you? Peace: I also join in praying for him. Me: oh ok

Me: So, tell me what it is like taking care of him on a daily basis?

Peace: Ok, when I wake up I do one or two things, then I will go there to get the peel to empty it because now that the catheter is out he urinates into a peel. Then I will give him the brush and tooth paste. He can brush his teeth by himself. I will go ahead and prepare his breakfast and then go and serve him. If my uncle is not around I will prepare his lunch and keep in a food warmer by his bed side but if my uncle is around I leave the food with him in the kitchen. On weekends I don’t go to work so I’m mostly around to take care of him. If he needs something he calls my phone. So I make sure my phone is always by my side wherever I am. Me: Apart from cleaning, how do you take care of him anytime he wants to visit the toilet since he is always in bed or in the wheelchair?. Peace: During the early stages of the condition, he could not do anything so I do it for him. What I do is, he always has a diaper on, so when it is soiled I remove it but when he started getting better he removes it himself when it is soiled. Then I go for it and discard it by wrapping it in polythene bag. Me: oh ok

Me: You earlier on mentioned that, when you are around you keep your phone by your side so that when he calls you will be there to assist him. If that is the case, tell me about your social life. Are you able to step out, for instance with friends, your beloved etc. especially when your uncle is not around.

Peace: Yes, but I’m not able to stay out for a long time and I don’t go far from home. Even during the initial stages I was not going to church at all because there was no one to takecare of him. But now that the condition is getting better, I’m able to go to church.What I do is I make sure I provide whatever he needs. Also, I took some days off from work to stay home and takecare of him.

Me: What about your personal relationship, was it affecting it in anyway. Because ofcourse if you cant go anywhere, and your beloved wants to see you, step out etc. What do you do?

Peace: So, as I said earlier, when I step out I don’t stay too long.

Me: I mean earlier on when the condition was more serious.

Peace: oh ok, then, I had to sacreifice….Me: that means your beloved understood the situation? Peace…laughing..Yes. Me: oh no, I want to know how things were between you during those difficult times. Peace: Oh yes, he understood, because if you are in this situation, there is nothing that you can do, Me: so he needs to understand…Laughing….ok

Me: Can you also tell me about your eating habits/patterns, sleep pattern at the time?.

Peace: yeah, this was in the beginning, for instance at night he can call for me to assist him change the position of his legs because it has being at the same position for a long time and it is causing him pain. After that then I go back to bed. Sometimes, the catherter must be changed, because when it stays in for too long it sometimes get blocked causing severe pain. So, at night he may call you to come and change the catheter. This results in sleepless nights, you can just imagine. Sometimes, I have to go to work the following morning. One more thing is that initially we were not told that the catherter must be change after some time. No one informed us about that, so he had it on for a long time and this resulted in pain. The catheter stayed in over a month and two weeks. When it should have been in there for just three weeks and then changed at the hospital. Me: eeeiiiish. Peace: If we had been informed earlier, i think it would have helped a lot.

Me: do you sometimes take something to help with sleep?

Peace: I realised that when I am stressed up, I start experiencing headaches, so i just relax and i am fine. So that is what I did, I try to get some proper sleep or sometimes just lie in bed not alseep but just to relax my mind and body. After sometime I am okay. Me: So, what happens when you go to sleep and he calls your phone and you are unable to answer, because if you are so tired and you say you sometimes try to get proper sleep especially when you feel you are too stressed up. He might call for your assistance and you might be fast asleep and you may not hear your phone ring. Peace: no matter how fast asleep I am, I always answer the phone. Me: Oh ok wow. Then it means the ring tone of your phone must be very loud. Peace: laughing..not really.

Peace: What about your eating.

Peace: Sometimes its difficult but I try to make sure I take in something because I don’t want to lose weight.

Me: initially, you mentioned that, the staff didn’t tell you anything about the management of the catheter. So I want to find out during daddy’s stay in the hospital, did any of the staffs, I mean the doctors and nurses, especially the nurses, take the pain to sit any of you down to explain things. Peace: No, the only thing they told us about was in connection with the administration of the medication at home and the date for review. Even that, he was the one who was told not us. Me: oh ok

Me: Do you sometimes feel overwhelmed with everything. Like you want to give up, you are tired of everything?

Peace: No, It hasn’t gotten to that. Me: how long have you been taking care of him, Peace: Since June last year, Me: Oh ok. But the condition has since been there over a year. Peace: yes.

Me: So, sometimes you get anxious and helpless especially when you don’t know what the future holds and what to expect. “ Eii adi ebe y3 yie”

Peace: Yes, since i am are a human being that taught will come into your mind. Me: Yes, so all these thought like what, I want you to tell me about them. Peace..Laughs, Yes, thoughts like, eii is it going to get better? It was coming during the earlier stages, when the condition was serious, I mean before the surgery but as we speak now it okay. For instance earlier on if he needed to do something, someone must do it for him. He couldn’t do anything for himself. But as time goes on and I see that the condition is improving all those thoughts started dissapearing from my mind. Even those the improvemnet in his health is slow, at least it helps encourage me a lot. Because I know we are winning. Unlike in the beginning, I was really very worried. I find my self always thinking about him, infact it’s the only though I have all day (Na ayem shieshi3 woho paa) I was always scared. Me: wow ok.

Me: Around here in Cape coast, what do people say when they hear that someone has cancer? Other places people have different perception about it. So depending on where you live or the community where you are coming from.

Peace: Ok here, when people ask what the condition is and you tell them it is cancer. They then to become very sruprise (Ey3 omu hu kakra). Me: I see Peace: Yes, but after telling them, they just don’t say anything about it again.

Me: So, let talk about how you raise funds to fort bills. You said you are three girls. So how do you manage, because I know it is a lot of money. Do you sometimes go for loans, do friends donate money or other things to help:

Peace: In the beginning I took a loan, because all my money was getting finished. Me: Are you the eldest? Peace: No, I have an elderly sister but myself and the other lady are twins. Me: oh ok, So do they support as well ? Peace: Yes, they do but because I am here with him I spend more money and other resources than they do. Me: Oh yeah, ofcourse.

Me: So, apart from the loans, what about your friends.

Peace: As I said earlier they help but I will have to pay them later. But for the loan I have been able to settle them. I settled them this past january.

Me: Is there anything you will want to ask that we haven’t discussed or any thing you would like to tell me. We have actually come to the end of our conversation

Peace: the nurses, some on them are good. Especially the male nurses. But for instance they need to educate us on how to care for them at home, like I mentioned earlier about the catheter issue. Again, we met a family friend who is a doctor at the hospital so, when we needed assistance or clarification we called her. She has been very helpful.

Me: ok. Thank you very much for your time. Godbless you

Participant: Grace

Explanations given, and annoymity assured and consent form signed

Interview lated for 18minutes/45 secs

The participant was interview at the genito urinary unit after the clinic around 4pm. She had come to the clinic with the father for a review appointment. We first met in the consulting room. Almost at the close of the clinic period. I then explained the study to her and her dad (patient). After which we agreed to do the interview after the clinic because it was more convenient for her.

Me: please can you tell me about you, in relation to your age, what you do, educational level

Grace: I am XX yrs, tertiary level, currently, Im not working because we are yet to be posted. Me: Ok, if I may ask, what programme did you do at the tertiary level? Grace: I did nursing. Me: which nursing school please? Grace: XX. Me: Oh ok.

Me: So who is the patien to you?

Grace: My dad

Me: So you said he has been living with the condition for a year now

Grace: Yes

Me: So you have been providing care for him for about?

Grace: Two months now. Me: So, previously who was caring for him? Grace: No one, he is alone. Me: What about your mother? Grace: They are no more, they are seperated.

Me: So, tell me since you took over, what exactly do you do for him on a dialy basis?

Grace: The care I provide has to do with his eating, personal hygiene needs. You can see that now he is weak, so i need to carry the water with the bucket to the bathroom for him, the main care is with the eating and ensuring that he takes his medications. Also, I take him on most of the hospital rounds, especially for the reviews and doing ivestigations and so on.

Me: I also know that this condition is associated with a lot of pains, Do you live together? Grace: yes Me: so what do you do when he starts experiencing those pains. Grace: My dad is alittle reserved. He will not complain until it becomes unbearble. So sometimes we buy pains killers, Me: What is the name of the pain killer? Grace: Lofnac or so..thats is if I am wrong. Me: So you buy it form the pharmacy? Grace: Yes. Me: Does it mean that the hospital do not provide you with pain reliefs. Grace: Yes.

Me: Would you say that the role you paly has affected you physically? For instance because of the up and downs, you have sleep and eating problems or perhaps find yourself always very tired. Tell me about your experience

Me: Since I started caring for my dad ive lost so much weight. I wake up at night, especially with my little girl also with me, I need to wake up very early and do some of the chores for the day otherwise she would not allow me when she wakes up. I will prepare their (my little girl and dad) food, wash and so on. Again, I also have my mind on him, so during the night the little noise I wake up to go find out what if everything is alright. So I cant really sleep well.

I find myself working through out the day(from morning till evening) so I get very tired. Caring for little children is hectic and then adding a sick person to it becomes even worse. My girl is 1 year. So, you can just imagine what im going through. Sometimes, you start doing something and then my dad would call for assistance with something else, so I will have to leave what I am doing and see to his demands. My girl will also want something and I will have to stop what im doing and serve her.So, to finish one particular thing it may take the whole day and I end up so exhauted by the close of the day. At night too I cant sleep.

My eating pattern, hmmm, I need to make sure my dad is served , my girl too is served by the time im done I would have even lost appetite. What happens is that, after preparing breakfast, I may decise that oh let me wash these few things, then before I know its lunch time, I get their food ready, and something comes up again, so I sometimes end up eating once and mostly in the evening.

Me: hmm, God will bless you. Because caring for a sick relative in addition to a child is not an easy task

Me: So how do you manage his personal hygiene needs, especially when he wants to visit the toilet?

Grace: Oh for that he is able to do it by himself but the major problem is with the urine, He is not able to control it. So by the time he gets the urge to urinate, the urine starts pouring before he could even get to the washroom. You know how urine is, if I don’t constantly clean the room, no one can even sit there. So I’m always cleaning urine especially with my little girl around crawling, touching and inserting things into her mouth. Hmm it is not easy for me at all. So, my day is full of activities. Activity after activity. So I end up always very tired.

Hmm, sometimes my baby will be at my back whiles I travel some distance to fetch water because there is not tap in the house. I have to fill a whole barrel. Me: Are you the only child, Grace: No, we are three. Just recently my sister died, Me: oh sorry… and that has even made the condition serious because he has been living with it for a while now but was fine until after my sister died. My other sister is not good news, she doest even care about anything so hmmm. Just as the saying goes (There is Mensah in every home). That’s my little sister for you. Me: Oh ok. But what happened to the one who died? Grace: Road traffic accident ooo. Me: aww sorry.

Me: Tell me about when you heard that your dad have Prostate cancer.

Grace: I asked if they were sure. Me: ok, so there was some kind of denial. Because, for me to thought that cancer can easly be diagnosed but my dad had gone round and round from hospital to hospitals and there are now diagnsing him of having the conditon.

Later on I became very sad after I came to the acceptance that, its true he has cancer. It was because of the fear and panic that I went into the state of denial.

Me: Do you sometimes get overwhelmed with everything that is happening?

Grace: Yes, look at mylittle girl now, she wont even allow me to talk to you. At home it is even worst, She will ask me to carry her, after some time, she will tell me to put her down. She wants this , she wants that. I have to also take care of my dad. It is not easy for me at all. Me: So, what do you do then? Grace: I excuse myself, leaving them both to sit somewhere all by myself to listen to music. I like music so anytime I listen to it, it relaxes me and calms me down. Other times I go to sleep leaving them by themselves. Me: You mean both the girl and your dad? Grace: Yes, what can I do? I am tired and exhausted.

Me: Ok, now what about friends etc, who encourages you through this though situation. Because listening to everything you telling me, means that you need people to really help you through. Grace: Yes, I have a friend and also my baby’s father encourages me a lot. They tell me very soon everything will be over so I should exercise patience and not give up on him. In fact , my dad has helped us. He did he’s best for the three of us so, I can give up on him now. One thing too is that, after my mum and dad got seperated, I had to stay with my dad so, im the best person to take care of him now. So sometimes I encourage myself, because if I were the one in his position now, he will be the one to take care of me and I know he will gladly do it. So, if the tables have turn and he is now not well, I am his child I must take care of him.

Me: What about church? Any support or encouragement from church?

Grace: The truth, we are not very spiritual, He will not even go to church if I opt to take him, but I try to attend service sometimes and I pray for him. Me: What church pls. Grace: Catholic church.

Me: Are u aware that the hospital has a counselling unit and have you patronised their services before?

Grace: Yes pls, But I have not been there before.

Me: So, where you live what comes into mind when you hear the name cancer?

Grace: Eeeii… in my place when you hear the word cancer it means that the person is going to die. Infact, you are waiting to die. Others don’t eevn want to hear the word at all. Others also ask if it is infectious. So I tell them oh no, then they will ask if you are very sure. So after telling them , you will notice that they begin to isolate them selves from you. You will notice that, this particular person you sit and chat with will now refuse to sit and chat with you always giving you excuses. Not once and not twice.

Me: So what about your social life, with your friends and the father of your girl.

Grace: ooooohhh, it has come to a stand still. I don’t go anywhere oo. Even myself im not able to make time in grooming. I have started keeping my hair natural because of that. Laughing…

Me: Tell me about the relationship between you and your husband now. Since you took up the caregiving role. Is he also living in the same house with you at the moment?

Grace: Hmm, its been very stressful, oh no it lives in Kumasi.

Me: So what do you do? How do you guys cope?

Grace: We talk on phone,

Me: What about your sex life?

Grace: hmm…even we talk on phone so ….laughing. Me: laughing

Grace: He is not happy about the situation, but because the person in question is my dad, he cant do anything about it. Because he cant voice out how his feelings, the slightest issues he takes offends. Sometimes, even things that does not even warrant that he gets offended becomes a major problem that we end up quarrelling about. Things we use to joke about now becomes an issue to fight about.

Me: You know he is not happy even though he hasn’t opened up to you about it. Have you tried to sit him down to discuss the issue and you both find a solution to it. You both can come up with something that can help your relationship in the regard. Have you tried doing something like that? For instance, he can come down from kumasi then you guys can meet up somewhere and enjoy some quality time

Grace: Yes we do that. But the slightest thing makes him offended

Me: So, please tell me were you given education on what is happening or even your dad? especially the nurses

Grace: No

Me: Can I say that you sometimes try to take something to help you ease the tireness, insomnia etc?

Grace: I really love music, so by 8/9pm when im done with everything in lying I bed I listen to music and it relaxes and calms me down.

Me: How do you manage the bills.?

Grace: My dad was working before this sickness, so he is using his savings at the moment. We havent gone for loans and also havent received any finacial support from anyone so far.

Me: Is there anything you would love to ask or tell me?

Grace: Yes, you suggest that in situations of this sort the patient and relations should be educated on what is going on. For instance, when we came, all we were told id he has praostate ca. Today too, we have just been told that he will have orchedectomy…Laughing. No one is explaining things to us. They tell you this big words without breaking them down for you to understand. Nothing on how to care for him at home and all that.

Participant : Joy

Explanations provided and ensured of annonymity. Consent form signed

Patient was met at the consulting room. Interview was conducted the same day at a quiet place within the hospital

Me: continues with explanation on the reason for the research.

Interview lasted for 22 minutes/ 13 secs

Joy: Madam, hmm, as we speak now, I have knee joint and waist pains. Sometimes I even want to give up. Because I am already tired of everything. My son used to bring him for reviews but I had to come today because he is a teacher and he needs to go to work. Today, as I was preparing to bring him for the review, I had to wake up very early, prepare wrm water to clean him up and dress him up. Look I have severe waste pain, I am even sick. It is very difficult ooo.Me: hmmm. Joy: he is also very weak now, he cant do anything for himself. So, if I’m working on him he cant help me in anyway, I will have to do everything all by myself it is not easy. Me: awwww… so when it comes to bathing, feeding, lifting etc you do it all alone. You must be suffering.

Joy: Look yesterday, I gave him a bath….Me..huh huh….so this morning I only prepared warm water to clean him up. By the time I finished with everything I was so tired. I was sweating profusely. And because the children are working, they can’t come and support/ assist me in caring for him.

Joy: I used to travel and bring plantain and other foodstuff to sell but because of the role im currently playing I’ve stopped. Currently I m not able to go to work again, my children have made a container for me but they are yet to fill it with items. There is nothing in the container, still waiting for them but for the mean time I sell gari, groundnuts and sugar in there. They said I should exercise patient. Me: don’t worry everything will be fine.

Me: Can you tell me how the condition all started?

Joy: It has been a year now. One day he told me he was travelling. But he didn’t go but rather came back home. So I asked why, but he told me he didn’t go where he said he was going but rather went to the hospital because he had recently passed blood urine and he knew that, I will panic when he tells me. That is why he did not inform me. At the hospital, a catheter was inserted.

Me: so, which hospital was that.

Joy: This place, this is where he always attended hospital.

He was operated upon by some white doctors some time back. That was a very long time ago, it was on the same issue, he was having difficulty in urination. And right now another surgery has been done again.

Me: So please tell, when you were told about the condition what came into mind

Joy: As for me, at the time I didn’t know how the condition is like, so when he told me I didn’t say anything about it. All I said was hmmm.

Me: So, whenever he is in pain how do you feel an what do you do about it?

Joy: ok, anytime he is in pain, I feel very sorry for him, I also kind of experience the pain he is going through. So one day he called one of his children, called Emma, that he should come and take him to the hospaital and that he was suffering. That same day, the son called the doctor, urologist through one of the nurses. It was then close to christmas time. So the son told me to bring him the next day, then a surgery was scheduled and was done the folloeing week.

Joy: hmm, madam, look yesterday the only money I had on me was eight cedis. Infact im not happy at all, I know what I get anytime I go to work or when I was doing my business but now I cant do anything because of this illness.

When I wake up in the morning may be he may request for tombrown, onn thing he like very much. Then lunch time I prepare what he want s to eat. One issue about him is that, if you prepare anything he hadnt requested he will not take it. So you must make sure you give him what he wants.

Me: oh ok. What about when he wants to go to the toilet?

Joy: I use diaper for him, so when he goes to toilet I wear gloves to remove and discard it. When im doing this I experience a lot of waist pain in the process.

Me: Are you able to sleep and eat well

Joy: I sleep and eat well. I don’t have any such problems. He knowas I usually get tired so when I sleep he does not bother me

Me: So, since he cant wake you up when you sleep, what happens to him when he is in severe pains

Joy: He doesn’t do anything. I remember one time he was in so much pain and heard him cry to God to end it for him because he was suffering. But I told him to stop and that when the time is up God himslef will end everything.

Me Oh ok. So during your stay in the hospital from the time he was diagnosed through to when he went through the surgery di any of the nurses take their time to exalin things to you concerbing the condition and how to care for him at home?

Joy: Not all

Me: where you scared at the time

Joy: yes I was scare, I was heavier than this, I have lost so much weight since this condition started. I have changed

Me: please what is your level of eduction?

Joy: I didn’t go oo madam. i stopped at stage 5, my mum was a trader so I was helping and I realied that it was lucrative, was making a lot of money. But I have regretted so much, so im happy my children went to school.

Me: So the work you do is that you are a trader?

Joy: Yes I am a trader

Me: please can you tell me your age?

Joy: XX years

Me: is there anything you would like to tell me or ask that we havent mentioned in our conversation?

Joy: What I would love to say is that, I need money to continue with my trading.

Me: So how do you fort the bills and other things? Do you go for loans etc.

Joy: Madam, my children are the ones who support, if we go for a loan how are we going to pay.

Me: What about your social life. Are you able to go for funtions when you are invited

Joy: oh yes, I go, Me: so who do you leave dad with? Joy: no one

When im going out, I clode the store. I leave him in the hands of God

Me: oh ok. Thank you very much

Me: so do you go to church with him?

Joy: I attend Pentecost and he goes to Anglican but he doesn’t go nad noe that he is weak he want to get a good standing with God. So I told the pastor, so the pastor will start comimg home to pray for him to accept christ.

Me : so do you get encouragement form people?

Joy: Yes, I get that a lot. From my children, friends, church members, cousins etc. Sometimes he is very annoying, he tells you to do this, whiles on it, he wants something else. He really irritates me sometimes but what can I do. I just want o give up sometimes but its not possible. He has taken care of our children, he live in our own house with abou ten extra rooms rented out. So he has tried for us, I cant leave him now.

Me: thank you very much.

Participant : Mercy

Patient and caregiver were met at the urology clinic. Explanation was given on the need for the study. Be rest assured that annonimity will be strictly maintained

Interview: lasted for 21minutes/20 secs at the quiet place within the hospital.

26/03/2019

Me: please can you tell me about your age?

Mercy: XX yrs

Me: please what about your educational level?

Mercy: Teacher training college

Me: What work do you do now please?

Mercy: I am on pension, but was a teacher before

Me: What is the relationship with the patient

Mercy: He is my husband

Me: How long has he been living with the condition?

Mercy: It has been almost 2 year

Me: Please can you tell me how the condition started?

Mercy: The whole thing was sudden, one faithful night he started complaining that he could not pass urine, so we had to get a taxi to bring him to the hospital immediately, so we took him to the XX hospital. It was there that we we told, the urine was stucked in the bladder so a catheter was passed to drain the urine and immediately he was relieved. A lot of urine was drained that night. He was really suffering. Me…hmmm…So how did you feel?

Mercy: I was scared, going up and down very worried.

Me: So, at the point when you took him to the hospital, how did the nurses receive you. Did they treat the situation with urgency or they were just sitted and relaxed.

Mercy: aww, the nurses ddi very well, they treated the situation with urgency. Immediately they called the doctor and put him in bed

Me: Please can you tell me what you do for him on a daily basis.

Mercy: Since this whole condition started, he is not able to sleep, so as the caregiver, I also find it difficult to sleep because if he is in pain or needs something and im fast asleep, I don’t know what will happen. Apart from that, things he used to do on his own, he is not longer able to do them without assistance. This situation has resulted in a lot of stress on the children and I.

Me: How many children do you have?

Mercy: We have five children.

Me: So has the situation affected the children?

Mercy: Im not able to make much time for them, but even that is not much of a problem because they are old.

Me: Please what is the age of the last child.

Mercy: XX years. Me: Oh k. The last but one has completed XX but still jobless so she is the one that assists me sometimes in taking care of her dad. One time, he bleed profusely one night and my daughter and I had to look for a taxi to the transport him to the hospital in the middle of the night. It was within that same week that he was operated upon. Me: Hmm…

Mercy: Another issue has to do with money, the little money we raise must go into this sickness. Either we are buying medications or paying for hospital bills and lab test.

Me: So, when it comes to the financial issues, do you go for loans, or you solicit support from friends or from your children.

Mercy: The children support us, we also go for loans. Because we are both on pension. So the salary is not adequate.

Me: Has your current role affected your social life in anyway.

Mercy: Now I don’t travel, I don’t go anyway. Even if there is a problem in my home town, I must force to return home the same day. I cant sleep out. Me..aww so it means it has really affected you. Mercy: it has really affected my life so badly. Me: It is well

Mercy: it is a very difficult situation, he cant sleep, he is always in pain, so you cant also sleep because every pain he goes through, I also go through the same pain. Im always tired. For instance, today I had to wake up at 4am and prepare everything then rush down to the hospital to get his card and things ready and also join the queue so that we can see the doctor on time. So I had to take the lead to secure the place whiles he join later otherwise even at 2pm we still would not have seen the doctor.

Me.. Ok. So how did you feel when you were told that dad has prostate ca.?

Mercy: I got scared. My heart skipped a beat. Me: why? Mercy; because it is a condition that kills. It also bring along pain and debt. Monies that can be used for something at home , we use in paying for medications etc. It has even turn him an old man at this tender age. Me: What is his age? Mercy: he is XX year, but he was a sports man that is why he still looks strong but this condition has changed him suddenly. He was a footballer. Now because of this condition, he has become weak all of a sudden. He can do anything, I mean anything at all. He has become weak in all areas.

Me: So when it comes to the sexual aspect please does it bother you in anyway. And how about him?. Have you tried to find out if he is okay about everything and not worried about the fast that he cant perform any longer so perhaps, you are doing something else somewhere.

Mercy: I reassure him all the time. I tell him not to worry. Even before the surgery he told me that the doctors said they were going to remove his testis. But the truth is that wont be a problem because we already have children, and we have even pass child bearing age. So I told him life is all that matters so we should go ahead to do the surgery. So he is fine. He doesn’t even think about it.

Me: So please tell me about the support systems you have.

For instance, sometimes you may feel like giving up. In such situations how do you cope.

Mercy: He is not able to go to church. But I go to church with my children to pray for him belief that the lord almight will heal him. I have never attended any counselling session, one one encourages me. I encourage myself everyday. This life has a beginning and an end, so it is all part of the end. So I belief that everything that is happening is from God. So I give him thax because he is still alive for me.

Me: oh ok…

Me: What education were you given at the time of diagnosis and when he was on admission. Especially the nurses.

Mercy: Before the surgery, we were given explanations were given on the condition, and also the things he will need for the surgery as well as how to administer the medications at home on discharge. That is all. No one took the time to explain to us, how to care for him at home as a caregiver.

Me: please do you take alcohol?

Mercy: No, Me: not at all? Mercy : Not at all.

Me: So how are you able to cope with all the pressure ad stress that comes with it.

Mercy: I try to plan by activities for the day, it really helps a lot. In so doing im able to make some time available to rest. And I don’t get things piled up on me.

Me: is there anything you would want me to know that we havent spoken or discuss?

Mercy: when we visit the hospital, there is too much waiting time. We come very early but the clinic do not start early at all. They must try and do something about it for us. Because we are already exhausted and some of the patients are also in pain and very sick

In addition I believe that as pensionaires some of the medications must be free, we have served the country our entire life. The national health do not cover most of the investigations. At our age, we don’t have money, the government must do something about it.

Furthermore, the nurses and doctors must explain well to us how to administer the medications at home so that we don’t administer them wrongly.

Date of interview: 19/04/2019

Audio length : 40minutes/53 seconds

Start of Transcript

Me: Thank you for participating in the study

Forgive: Oh it is ok

Me: Please just as I have explained to you earlier. This study is to find out what caregivers go through as a results of the role they play. Rest assured that it will be annonymised. Consent form signed

Caregiver and patient were both met at the urology clinic after they had seen the urologist and made aware of the need for admission for surgery. Both patient and caregiver were provided explanantion on the need for the study and they agreed to take part. Patient was then sent to the ward for the admission process after which the interview was held later on within the facility, specifically around 4pm at the XX snack bar after the bar had closed.

Me: Please may I know your religion?

Forgive: I’m a christian, Jehovah witness

Me: Please what about your age?

Forgive: XX yrs

Me: Oh ok

Me: Please, who is the patient to you?

Forgive: He is my husband

Me: Please what is your level of education?

Forgive: Form 4 (secondary)

Me: As for us the young ones, we don’t understand this form 4 and others. Both laughing….

Me: Please you mentioned that it has been a year since he was diagnosed with the prostate cancer

Forgive: Yes, it has been a year now since he was diagnosed with the cancer.

Me: ok

Me: So, please are you the sole caregiver of the patient through out this one year?

Forgive: You mean, when it comes to monetry aspects? Me: oh no, I mean everything that has to do with caring for him.

Forgive: Yes

Me: Please do you have any children?

Forgive: yes, we have four children, they are all adults now.Me: ok, please tell me more about them.

Forgive: The first born is married and living with the husband now in XX. The last born is also married, she even came with us this morning to see the doctor. She has gone home now, but will return soon Me: Oh ok. Forgive: I also have two boys but they are not married. So the elderly boy is living with us at home.

Me: Please can you tell me how the condition all started?

Forgive: It statred on faithful night around 2am, when he started complaining of not able to pass urine. So this continued till afternoon, and we realised that it was getting worst. So, we took him to the hospital. When we got to the hospital, a catheter was passed right away to drain the urine. The doctor told us that he has prostate enlargement. So, the catheter stayed in for two weeks after which we were told that it will be removed. So when the rubber was removed, I thought that was it. But afterwards a request was given for us to do PSA. After the test, he was put on injection. The injection was 1500 ghana cedis and was to be taken for 3 months.

Me: So, were you told why the need for the PSA?

Forgive: Yes, we were told that, the PSA is going to be done to find out if there is cancer in the prostate. So, after the results came out, they said it was prostate cancer.

Me: Oh ok. I see

Forgive: After the test, he was put on injection, which is 1500. To be taken evey three months for. But we were able to take the injection twice, but on the third time, we had to come on transfer to Cape coast from Accra. So he told the doctor back at 37 hospital that we are going on tranfer and that he wont get the money to continue with the treatment so if there can be an alternative, he should help us out with that. But the doctor said there was no other alternative. So we should go and return to continue the treatment when we get the money.

Me: Please before we continue, I would like you to tell me how you felt when you were told that he has cancer

Forgive: I was very scared. I was really afriad, especially the day he could not pass urine and we went to the hospital. Because I have seen how people who have the condition suffer and what they go through. For me when the rubber was inserted, I told it will stay in forever so it got me very scared.

Me : So, it means that the insretion of the rubber was what got you scared and afraid but not the fact that he has cancer.

Forgive: Both the rubber and the diagnosis of prostate cancer got me scared and afraid. Yes that condition.

Me: So, anytime you hear that some one has cancer, what comes into mind?

Forgive: I heard that, there are different types of cancers, I had of breast cancer, stomach cancer and throat cancer. But I don’t really know how it looks like. Me: ok. Forgive: I also know that, people say it is a condition that is very painful. I see breast cancer on TV.

And also, one of my grand mums had one of her breast cut because of breast cancer during her adolescent stage, but she grew very old before eventually dying, even she didn’t die from the condition but old age. She however had all her children despite having one breast.

Me: Ok

Forgive: So, when I was told that he has cancer I really was scared and afraid because when I look at the stress and pain he went through when he could not pass the urine, hmm sister. When we went to Lekma, there he tried to urinate but when he was offered the container, he comes back he there is nothing in the container. He tells me he still cant pass the urine. We could see that he was suffering. I really felt pity for him, because he was going through a lot of pain. At XX, the doctor we met asked us to go home and return the next day, he didn’t even give him any pain relief. I was surprise, because the man could not pass urine and it was obvious that he was going through so much pain and yet nothing was done to drain the urine. So my son called a friend of he’s, who asked us to go to XX immediately, when we got there, the doctor did very well. It was there that the rubber was passed and the urine was drained before my husband was relieved. So I really felt pity for him. Me: Awww…hmmm. Forgive: So, after everything we came back home, but at home I realised that he gradually started getting weaker, he could not take care of his personal hygiene needs especially bathing.

Me: So please can you tell me briefly what you do for him on a daily basis?

Forgive: oh k. For instance, in the morning he will want to visit the toilet but he can’t use the public facility so he will have to go to toilet in a pot then I make sure I discard it. This is because we don’t want the dressing to get wet and also, because of the fact that our apartment is a chamber and hall with kitchen and a rented one.

Me: oh ok, so, even when it comes to bathing, you will have to do it for him

Forgive: Yes, and when it comes to his food too I take care of it. But my major problem is the toilet. Because if he needs go to toilet three times, I have to make sure I go for it afterwards to take it to the mains to discard it. For the bathing I have a big basin in my kitchen, he sit in the basin and I clean him up.After which I pour out the water. Then I go ahead to do other things for the day

Doing these things are not easy, I have waist pain and I find myself always very tired.The waist problem was there but was not as serious as it has become now, and it is all because of the role I currently play.

Earlier on I made mention that, he told the doctor that finacing the treatment is now a problem so if there is an alternative he may want to try that, but the doctor said no, and that he should get the money and return to continue with the treatment. He then decided to start herbal treatment at home, it helped with the pains to some extent but was not very effective but there was difficulty with urination. He continued with the herbal medications despite there was no improvement in the condition.he tried about four different herbal facicilties. Suddently, he took ill of smalaria, so we went to XX hospital, but there was no improvement. So, one of his bothers took us to XX university hospiatal for further management. It was there that the doctor asked if he had ever been diagnosed with a particular condition apart from the malaria he currently had. He then had to expalin everything to the doctor, and the doctor advsed that we come to the cape coast regional hospital because there doctors here who are specialised in the case of prostate. We were then givena transfer to this place, and we saw the doctor. Treament commenced, so he use to come for revie every two month and when we check the PSA the values were decreasing hence the hope that evrything will be fine. But suddenly the values started increasing so the doctor said he will have to undergo a surgery or will start the previous injection he use to have back then in accra, but this time, it is no longer 1500 but 1800 every 3 months for two years. Operation, was another option of which we are expected to pay 1000 ghana cedis, apart from the medications that will be used before, during and after the surgery. Me: Eiiii, that is also expensive. Forgive: Also, with the first treatment, complications that may occur after may be that, he could have kidney failure, paralyze or may become impotent. This is what we were told by the doctor. So, after we got home I had to find out from him, which on the treatment would he prefer. He said he will rather opt for the surgery, but now the next issue has to do with how to raise the money for the surgery. Me: oh k.

After a while when raising the money became a problem, he decided to go back and continue with the herbal treatment.

Me: So, when he started the herbal treatment again was there improvement?

Forgive: No oo. Me: Hmmm

Forgive: But suddently he started complaining of waist pain, which he describes as very severe. He’s walking changed, he was now drawn more to the affected side of the waist. He compalined to the herbal clinic doctors and different medications were given for the pain yet there was no improvement, rather became even more serious. It drew worse and worse, he cant sleep anymore, not at night and even during the day. The pain so sereve, that the muscles of his buttock that he cant even sit with the pain radiating to the lower limbs. This continued for sometime, as he continued to buy all the herbal medications he comes across. Me: Ei..

Forgive: Because of the situation, I cant also sleep as the pain is too much for him to bare and also the pain makes him grinding on his teeths which you could hear so loudily. Me: wow

Forgive: The truth is the herbal hospital did not tell us that it is the cancer that is causing all the pain and yet kept giving him medications. Any time we visit, they come up with something different which according to them is the reason for the pain. Sister, because of this, even after cooking I cant eat, I’m always worried and afraid because I don’t know what this is leading to anymore. So, one day I asked him that, looking at what is happening now, what are we going to do about it? So we called a newphew of he’s, who is a doctor. It was then that he was told, the pain was a results of the prostate cancer. Me: Ok. Forgive: Weeks after he has been told the cause of the pain and asked to go back to the regional hospiatal to see the doctor he was still adamant.

Me: I believe its because of the money involved.

Forgive: oh sister, I don’t think so. Me: Why do you say that? Forgive: Because, anyways I can agree with you to an extent, but the 1000 gh cedis could have been raised. Me: Yeah, you are right. Because I also know that herbal treatment is very expensive.Forgive: Yes, anytime he goes to the herbal hospital, he spends between 500 to 600 gh cedis, each time he goes there. Me: Really? Forgive: Yes.

Forgive: I really didn’t understand why he would want to go have the surgery. Probably it may be because of the cut that will be made or because of the fear of becoming impotent after the treatment. I cant really tell sister. He once told me that, he has heard people say that the orthodox medicine can cause kidney problems. So, he will rather try the herbal medicine. Me: Ok

Forgive: Sister, I tell you, he cant sit oo. It was just last week Monday that he finally agreed to come and see the doctor. Immediately we got here and the doctor his eyes on him, straight away we were asked to be admitted and he was told that it is the prostate that was causing all the problems.

Me: oh ok. So now has he agreed to undergo the suregery?

Forgive: Yes, but he was given some medicines for the pain after which the surgery will be done and within three days after taking the medicines the pain went down. Me: Good, we bless God.

Me: Tell me about your experience in the hospial.

Forgive: Hmm, as I told you earlier. I have a waist problem. When we arrived here earlier this morning, I had to sit down immediately because I was in so much pain myself. Mostly when we come, I have to do all the errands, go here, go there, and also wheel him around all by myself so this time my son was home so we came with him. Formally there are orderlies who help relatives with wheeling of patients but now they don’t do it anymore. You can just imagine that, if I had not come along with my son what was I going to do, because I couldn’t have wheeled him around in this state.

Me: So you see, people who take care of sick people at home go through a lot, but they don’t say it so they cant be helped.

Me: So can I also say that it has affected your social life?

Forgive: During the earlier satges of the condition, I’m able to go for programmes and functions. My husband is a driver, he was able to work at least three times in a week during the earlier stages so when he leaves I also go but now that the condition has become severe, he is not able to work anymore and I have to takecare of homa at home.

Me: Please what about your sexual life? Is it a problem?

Forgive: It is not a problem at all, Me: What about him?.

Forgive: Oh no, the other time his nieces and nephews were even teasing him with it. He told them that, now the strength is not there but if it was there why not. Moreso, he has given birth to all his children so that should not be a major issue now. The most important thing is for him to recover from this condition.

Me: Please did any of the staff especially the nurses take the time to expalin to you what is expected of you as acregiver at home.

Forgive: No one educated me on how to go about things at home, and because of that, it is really a problem, as I find myself in a fix, not knowing what to do for him sometimes especially when he is in pain. But we were told how to administer the medications. No one mentioned anyside effect that may results from adminitration of any of the medications.

Me: Have you ever thought of giving up.

Forgive: oh not at all. I only pray for God to heal him

Me: Do you sometimes think that the condition had resulted from some kind of sin he has commited?

Forgive: Oh not, but ofcourse may be as a results of so many things, currently the world is being ruled by the devil, so anything can happen. Someone may contract a condition as a result of something done in the past, others, from their enermies and so on. When we went to XX hospiatal, I asked the doctor to tell me the cause of the condition, but he mentioned that even as a specialist all research done have not really clearly come up with what the cause of this condition is.

Me: Please have you ever met a counsellor before, or go to see your pastor or seek encouragement from?

Forgive: No, I have never met any counsellors before, And as a Jehovah witness, we done go to seek encouragement form pastors and what no. We don’t even have pastors. As memebers we share our experiences and others learn from them.

Me: So financially how do you cope?

Forgive: My children help, and also the little saved up by my husband. At church too, support is provided for those who need it but we have not asked for help from any of those avenues or even taken a loan.

Me: please is there anything you would like to tell me or ask?

Forgive: It is my prayer that, caregivers will be considered and appropraite help can be given.

Also, for the hospital and the staff, I know with every work there is time to start. We must report at the right time because patients come to the opd and wait for a long time before they are seen, the staff must also watch how they relate to the patients and their relatives especially the nurse.

Interview date:15/03/2019

Participant: favour

Interview lasted for 17minutes/53 secs

The interview was held at the XX after caregiver has been provided explanation on the importance of the study about a three days earlier. Anonymity assured afterwhich the consent form was issued to be signed on the day of interview.

Me: please, what is your age?

Favour: XX years

Me: please what is your educational background if I may ask. I mean were you able to go to school to some level?

Favour: yes please, I did.

Me: To which level please?

Favour: stage four (primary)

Me: So, please what do you do for a living?

Favour: I am a trader. Me: what do you sell? Favour: food (Etew)

Me: Please who is the patient to you?

Favour: My father. Me: thank you

Me: So, as I discussed with you about three days ago, im here because of that discussion we had.

Me: So, how long has dad been living with the condition?

Favour: About a year now. When it started he used to complain about pain around the lower abdomine. But there is this retired doctor friend of he’s whom he used to see during the early stage of the condition. He also used to say that anytime he tries to pass urine he gets the feeling that everything does not come out.

Me: So how long have you been taking care of him?

Favour: Since the condition started. About a year now

Me: What can you tell me about the condition?

Favour: I cant really say much about the condition.

Me: Ok, can you tell me if anything has changed about your dad since he was diagnosed with the condition?

Favour: Before the condition started, my dad was working, now he can not work anymore. He is always at home. Me: Is he able to walk or you have to always assist him? Favour: No, he is able to walk, because he uses the walking stick.

Me: Do you know what condition he is suffering from?

Favour: They say it is cancer, the doctor mentioned that cancer has been found in his genitals.

Me: oh ok

Me: What are some of the things you do for him on daily basis?

Favour: Hmm

Me: I mean for instance, who cooks for him, bath for him, etc.

Favour: My mum cooks for him, as for bathing he is able to do it for himself.

Me: Do you live with him?

Favour: No, but I go there everyday to take care of him because my mum is also not well.

Me: So, how long do you spend there in a day

Favour: I go there early in the morning, and mostly return home in the evening. I spend almost the whaole day at his end.

Me: So how has the care giving role affected your eating, sleeping pattern etc

Favour: It does not affect me much, because even if it does, I know im suffering for my dad so it is not an issue.

Me: Oh ok. I know you are suffering because of the care you give to your dad, and that it is not a problem for you at all because it is your dad. But as I explained to you before we commenced the interview, I want you to tell me everything you go through to help so that people like you can be assisted.

Favour: Ok, as a results of the role I play, I always find myself very tired and I am unable to sleep adequately so I sometimes take sleeping peels to help me sleep.

Me: what medication is this.

Favour: Quick action, Me: eii, it means you take it a lot. Favour: I like it because they say it helps relief body pains, and it is good for me anytime I take it. I even have one in my bag as I speak to you. Me: eeeeiii…ok

Me: So tell me about your eating pattern?

Favour: Sometimes I eat once a day, because I even forget to due the caring role.

Me: What about the finaces

Fovour: Infact, I worry a lot about the finances. Expecially when it comes to settling bills and purchasing medications. I call my other siblings then we all contribute to take care of osme of these things. Sometimes it becomes a problem, expecially when we cant raise the money. It becomes frustrating and we don’t know what to do.

Me: Please are you married, do you have children?

Favour: Yes, I am married with children

Me: So, are you able to make time to take care of them in addition to the current role you play

Favour: Yes, I prepare them for school ,then I proceed to my dad’s place

Me: So hwoa re the children taking the situation, do they complain that you are not able to make time for them because of grand paps condition

Favour: Not really because they know that there is nothing that I can do about it. He is my father so I have to take care of him

Me: What about your social life?

Favour: Im not able to go for programmes, especially when he is not doing very well. I don’t also have friends , so that not much of a problem for me

Me: What came into mind when you were told that your dad has cancer?

Favour: Eiii..me yem shi me (Heart skipped a beat). Me: Why?

Favour: Because I know it is not a good ilness, it mostly kills its victims.

Me: So did the doctors and nurses explain to you all about the conditon.

Favour: yes, they said if treament is not commencesd immediately it will turn him into a vegetable. Also, if he develops an openned wound, he will smell all over. So we must make sure we start the treatment. So, that got me even more scared because if he becomes a vegetable and starts smelling I will still go and take care of him, and that will be very difficult.

Me: Were you educated on how to care for him at home, especially by any of the nurses.

Favour: Yes, we were taught how he was goig to admister the medication. That is all

Me: Are you sure please? Favour: yes

Me: Please so when things become difficult and you cant rasie money for the treatment, do you go for loans.

Favour: No please. We always try to contribute to pay for the expenses.

Me: So, when you become overwhelmed, how do you encourage yourself

Favour: Most of the time I encourage myself, family members encourage me a lot. But I have any seen any counsellor before I didn’t even know that people like that exit in this hospital.

Me: Is there anything you would like to tell me or ask me?

Favour: At the moment, we are in need of finacial support. We have spent all our monies and saving on this conditon. My children are in school and I need to pay fees. Please it is not easy.

Me: ok.. everything will be fine. Thank you very much for your participation.

Participant: Humble

Date of interview 19/03/2019

Duration of interview: 25 minutes/18 secs

The prostate cancer patient was met on the surgical ward, after approaching him and the relevance of the study explained, the contact of the sole family caregiver was collected. The caregiver was contacted, met in person and the relevance of the study again explained to her, after which she agreed to undergo the study. The interview was scheduled in three days. The interview was conducted on the hospital premises because patient was still on admission and caregiver sleeps in the hospital.

Observation made: caregivers sleep under unfavourable condition within the hospital.

Upon agreeing to participate in the study, a consent form was issued to be signed/ thumbprinted.

Me: Please who is the patient to you?

Humble: My husband

Me: Earlier on you mentioned that you sleep here in the hospital. May I know why, and if the hospital have provided a place for relatives to sleep when they visit?

Humble: I sleep here because of the money involved in going home and returning everyday. My is abit far, so If I have to always go and return is will be very expensive. I live at komenda.Again, the hospital have not provided a place for us, we sleep outside on the floor and we a lot of mosquito bites.

Me: Please how long has he been living with the condition?

Humble: For close to a year now. We came here and a rubber was passed into his genital because I had difficulties in passing urine. After that he was checked and the doctor said they will do operation for him. But we were not able to raise the money in due time, so, it is now that we have been able to get the money that is why he has been admitted. Me: okay

Me: Please are you the sole caregiver of the patient?

Humble: Yes please,

Me: What do you do for him on daily basis and even her in the hospital?

Humble: Here in the hospital, I do most of the errands, especially with buying of medications. At home I do everything for him, from cleaning him up and helping him if he has to go to toilet.

Me: Looking at the role you currently play, has it affected your eating habits in anyway?

Humble: Hmm as for the food aspect, it is by the grace of God. Sometimes friends give us something little to manage with. I am a farmer but since the condition started, I havent been able to go to work. So, life has become very difficult. Me: hmmm, it is well.

Me: What about your sleep pattern, has it affected it in anyway?

Humble: Sleep is a thing of the past, at the moment what I do best is worry, and worrying deprives you of your sleep. When I’m constantly worrying about his health, how to raise money for the medical bills, buy medications and pay for laboratory investigations how can I sleep? Currently, the life we are living now, it is only God ooo Me: hmmm, everything will be ok.

Me: So, sometimes does it happen that, you get so tired that you are not able to do anything?

Humble: yes it does happens, because of the role I play currently, I find myself always tired, coupled with lack of sleep. Sometimes there are things that needs to be done, but because of the exhaustion I’m not able to do them.

Me: Do you sometimes get scared or afraid as a result of the fact that your husband is suffering from this condition?

Humble: Hmmm, when he is always complaining of pains in different parts of his body, for instance pain in the penis because of the rubber that has been inserted, pain around the waist, pain in his abdomine, etc. Why wont I be afraid and scared. At night when we enter into our room to sleep, we don’t sleep till the next morning, because of persist complain of severe pain everywhere with me not having any idea what to do to help him. If you live with someone suffering from a condition like this you will never be ok, you will always be worried.

Me: So, can you please tell me some of the things you worry about apart from how to foot medical bills etc.

Humble: I worry about what we are going to eat, since im no longer working, because I cant watch him starve. If I don’t take good care of him and something happens to him people will say that I caused his death, meanwhile we both don’t have money, but I have to try and make sure he is ok, even If I don’t get to eat and he has something to eat then I am okay.

Me: How has the role you paly currently affected your social life?

Humble: I am not able to go anywhere since the condition started but my friends and family know the current situation I am in, so if I don’t show up at gatherings it is not a big problem.

Me: So, what work do you do?

Humble: I am farmer. Me: oh ok

Me: Are you able to go to work?

Humble: Since the condition started I havent been able to go to work, I pray that God will heal him so that I can go back to work.

Me: What comes into mind when you were told that he has cancer?

Humble: It was the doctors who said that he has cancer, as for me I don’t know much about it.

Me: What preparation were you given for you to be able to care for him at home. Were you educated on the role you are currently palying.

Humble: They educated us on how to administer the medications. But nothing else.

Me: So, when he is in pain what do you do about it?

Humble: We were given some medications to be administer, but when they finish we were asked to get more from the pharmacy.

Me: Please, you have complained a lot about your finances. I want to find out how you are able to raise money take care of him.

Humble: The children help us a lot. I have one child with him but he has other chidren and they are adults and working. Also, friends and the church support us a lot. We have never taken a loan.

Me: In case you become overwhelmed with everything, how do you encourage yourself. There is a counselling unit hee in the hospital, have you ever visited the unit before?

Humble: I have not visited and counsellor before but the church and sometimes friends are the people who encourage me to keep up with the good work. Sometimes I encourage myself.

Me: Do you take him to churches for him to be prayed for?

Humble: Yes I use to but since he became more ill I havent been able to anymore, but sometimes the pastor comes home to pray for him.

Me: So, how are you able to do everything as you are suppose to within the day?

Humble: I plan my day before day break, so I may say I plan the day ahead the day before. So, before I wake up in the morning I already know I what I need to do within the day

Me: So, do you think doing that has helped you a lot?

Humble: yes to some extent, because I get to do all that I am suppose to do for the day only that sometimes I get very tired.

Me: So, please what are some of the resources that has helped you within this year that you have been taking care for dad.

Humble: I told you we are both not working so my children are the people who support us but some friends comes along with some food items.

Me: Please as we a bringing our conversation to an end. Please is there anything you would like to know or say that has not been mentioned in the course of our converation?

Humble: What I will say is that, we are open to any help/support form anyone. We will be very grateful to the person.

Me: ok… thank you very much for participating in this study.

Participant: Passion

Date of interview: 15/03/2019

Duration of interview: 32minutes/ 2secs

Patient was met on the surgical ward on admission. I approach the patient we had a discussion on the study and its relevance. I then went ahead to collect the contact of the caregiver who was said to be around the hospita premises. The caregiver was then contacted and after some few minutes we met. I explain the study to her, as well as made its relevance also know to her. She then agreed to participate. The interview date was scheduled to three days from the actual meeting day at the hospital premises were the caregiver currently stays because her home is far from the hospital.

On the day of the interview, explanations on the need for the sudy was given again, after which caregiver was offered a consent form to thumbprint. Anonymity assured. That day, her daughter was also present and insisted that she will be part of the interview even though her presence was not needed.

Observation made: The daughter made the caregiver uncomfortable as well as the interview unecessarily long with irrelevant information. So in this transcript only the few relevant statements made by the daugther will be included.

Me: Please may I know you age?

Passion: Age mentioned in the local dialect, Me: Did not understand, so daugther mentioned in english as XX years. Every body laughing….

Me: Educational level please?

Passion: Did not school at all

Me: Please what do you do for a living?

Passion: I am a farmer

Me: Please who is the patient to you?

Passion: My husband

Me: ok

Me: How long has dad been living with the condition?

Passion: It has been about three years now. It started with difficulty with urination, so a doctor told us that unless they do surgery for him. But before then he was told that a rubber will bw passed to help with the urine but he inially he refused but later on he agreed because he was in severe pain. The rubber stays in for months then it is removed, but anytime it is removed the pain become very severe and unbearble for him. So, we went back to the doctor and we were told that, it is important that the surgery is done otherwise the pain will continue like that. So he called his children to inform them about what the doctor told him. Before then he had gone to see another doctor in XX. Me: Is it a hospital? Daughter: yes, a mission hospital. Me: Oh ok. Humble: according to him the doctor at the mission hospital said the surgery to be conducted will be in two parts and that is going to cost 2600 ghana cedis. So he prefers to have the suregery there than come to XX. Me: oh k. Passion: Raised the money and sent him for the surgery. The whole proceedure was successful. He passed urine normally with no pain, and we had even gone to church to thank God. Me: ok. But, suddenly, one of his limbs felt very cold and became swollen. Eiii what is happening. The other limb also become swollen and both limb became very hard. So, we took him back to the mission hospital, several investigations were made. So, later on the doctors there gave us a referral letter to XX again. The doctor requested for PSA to be done which came out high indicating that it is prostate cancer.

Me: How long have you been taking care of him

Passion: It has been a year now since he became very ill. I say this because earlier on even though he was not well he could go about his normal daily duties with my assistance but now everything must be done for him.

Me: On the daily basis what do you do for him?

Passion: When we wake up in the morning I clean him up, then I find out from him what he will prefer for breakfast. The truth is he could not eat much especially when the condition became serious. I do everything for him, if he needs to go to toilet I have assist him and later discard it. It is not easy

Me: So can I say the role you currnetly paly has affected you physically, especially because you said it is not easy. What is not easy, tell me more about that.

Passion: As for sleeping, hmmm it is a problem. I am not able to sleep. At home when when enter the room in the evening, he wont sleep for you to also sleep ooo. He constantly complains of pain, turning and tossing on the bed, so cant also sleep. Some times this continue till morning, other times by 3am you find him sleeping then I am also able to get some sleep. Sister I am really suffering. It is not easy at all. As for the food I am able to eat.

Me: Do you get worried as a results of his current state,

Passion: As for worrying, hmmm, it is one thing I do, especially went the limbs got swollen and he starts experiencing the pain. It really gets me very worried.Because when we got married this was not how he was and all of a sudden this. Sometimes I don’t even know what to do to help him. Me: hmm. Passion: Sometimes, I do everything I have been told to do yet there is change and improvement in the condition. It is very frustrating.

Me: It is well

Me: please are you able to go to the farm

Passion: No, now I am not able to go to work anymore since the condition got serious.

Me: Are you able to go for family functions?

Passion: No, I’m not able to anymore. Because we live alone, I don’t have anybody staying with us who will take care of him when I am away. So, I have no other choice than to stay home by his side.

Me: So, when you head that dad has cancer, what came into mind?

Passion: What I know about cancer is that, it is a wound that develops inside that body, so when you are put on medictaions for some time you get cured and that is all. But when we came here, we were told that he was going to have the surgery because of the cancer and that really got me worried. Meaning, I was wrong about the condtion. I also heard that it can actually kill and the fact that my husband is going through so much pain because of this cancer is also something that tell me that cancer is much more serious than I thought.

Me: Please can you tell something little about how the doctors and nurses, especially the nurses relate to you. For instance, are they nurses deucating you on the condition and how to take care of him at home after discharge.

Passion: We were told that prostate cancer is the reason for all the pain he is going through, I was also told about how to administer the medication at work.

Me: Apart from the above, what else were you told?

Passion: No one educated us on any other thing apart form what I have mentioned

Me: Please, so how do you raise money for footing his medical bills and other things.

Passion: He has a cocoa farm, so we harvest some of the cocoa and sell to take care of the bills. Sometimes the children also support.

Me: Please how do you encourage yourself when you become overwhelmed with everything that is going on?

Passion: I have never sought counselling services anywhere, I didn’t even know there is something of that sort in this hospital. But the church and friends encourage me a lot, to never give up

Me: When you were informed about the condition, what was your reaction?

Passion: As for me i didn’t know much about it, so, I accepted everything I was told.

Me: Oh ok

Me: Please is there anything you would like to know or ask me that we have not mentioned in our conversation?

Passion: All I’m praying for is for God to heal him.

Me: So, do you take him to churches to be prayed for, because you believe that God will heal him and as a Christian it is your prayer that he is healed

Passion: No, I don’t take him from church to church, my church does not allow that. We pray for him in my church.

Me: Please, what about your sexual needs?

Passion: It is not a problem, because we have all our children now. As for me I am there, but now he his the one who do not have the strength anymore to have sex.

Me: But please do you sometimes discuss with him, to find out how he feels about it. Because it is also one aspect that bothers them a lot.

Passion: I haven’t, but will try and talk to him about it as you have said.

Participant: Kindness

Date of Interview: 20/03/2019

Duration of interview: 40 minutes/ 47secs

Patient was first met at the urology clinic, on his review visit. He was handed over to me by the doctor after he was done seeing him. At the consulting room, I introduced myself and engaged him in a little chat on what the study was all about and its relevance. In the course of the conversation, I realised that he had reported to the hospital alone. The caregiver is his wife, who is back at home. I then took his contact and that of his wife.

Later on, in the course of the week I called to schedule a date and time that will be best convenient for the caregiver so I can visit for the interview to be conducted. Patient and his wife live at Komenda, so, I went all the way to Komenda, where we had a successful and interesting interview.

The interview started with a prayer from the patient himself. The reason for my visit was made known to them once again especially the caregiver (wife). Explanation provided on the relevance of the study. Anonymity assured and consent form signed

Me: please can you tell me your age?

Kindness: XX years

Me: please what do you do for a living?

Kindness: Please I am a trader

Me: Your educational level please?

Kindness: Tertiary (Commercial school), could not further because there was no help so I later started working with Komenda sugar factory. After which I moved to XX. Me: Oh ok. Why was dad in Liberia? Kindness: Yes. Me: wow. Kindness: You must have chilled paa oo. All laughing

Me: Please who is daddy to you?

Kindness: He is my husband

Me: How long has he been living with the prostate cancer?

Kindness: It should be about 4 years now, but when they finally told us that it is prostate cancer, it has been 2 years now.

Me: So, please have you been the sole caregiver of daddy at home all through these years?

Kindness: Yes

Me: Please can you tell me something little about how the condition all started?

Kindness: When the condition started, we could not really tell what it was. So, someone directed us to a hospital in Accra. Me: which hospital is that? Kindness: XX. Me: oh ok. He spent a lot of money there, about 3000 Ghana cedis. He continued taking the medications given to him by the hospital but there was no improvement. So, after a while he decided to visit the Cape Coast Teaching hospital, and that was where the actual condition was revealed to him. Me: So, tell me about the treatment that was given to you at medi moses, was it effective? Kindness and patient: they both answered, it was not effective, was total waste of money and resources. Me: Oh ok

Me: So, on a daily basis, what do you do for him?

Kindness: What I do for him mostly is cook for him, he is able to take care of himself and even sometimes assist me when I’m cooking. When it comes to administration of his medications, he is able to do everything by himself. Unless the medication is a herbal medication, then for that I need to cook it for him.

Me: If I may ask, has the role you play affected you physically in any way?

Kindness: So, far he’s had two operations. To be frank with you, it was during the first operation that I suffered a lot. Because of the surgery he could not do anything for himself. I had to cook, wash, clean him up, lift him and so on. It was a very serious experience. I didn’t get it easy at all. But currently he is okay, so he can take care of him self without much assistance form me.

Me: During the time he had the second surgery, were you able to make time for work?

Kindness: At the time, I was doing feeding programme, I was able to do everything for him, especially his food before I leave for work. Me: oh k, don’t you get very tired. Kindness: Yes, but he was very co- operating so I was ok.

Me: So how did you feel when you were told that he had cancer and needed to undergo surgery?

Kindness: I got very worried, but because of God I knew that everything will be fine. Also, money was another major problem. It was our prayer that, God will send help, so that we can fort the medical bills and other things. God has been faithful.

Me: So, through out the difficult times, you mean you prayed to God for help and at the end of the day everything was ok.

Me: So how has it affected your social life? Are you able to go for functions?

Kindness: Yes, I was able to go anywhere I wanted to go, especially church. There is this little girl who was staying with us. So, if I prepare everything down, I can leave. She is only there to go on errands if he needs someone send to do something for him

Me: It means that you always plan you days ahead, so that you can be able to do everything you want to do. Kindness: Yes, exactly, I plan everything. For instance, this morning he has eaten, I have prepared his lunch. So, after talking to you I will set off for work. By the time I return in the evening he would have had some tea already. So, when I plan it helps me a lot

Me: oh ok.

Me: The day you were told that dad had cancer tell me how you felt?

Kindness: The day I heard that news I was really worried? Me: why? Kindness: Because some people say it is infectious, others also say it kills its victims within a short time. So, as I grew more worried, I began praying the more, committing everything into God’s hands. Because I don’t have anyone, if he dies and lies me life will be very difficult for I and the children. So, I always pray for God to bless him with good health and long life. And even when I’m worried, I make sure he does not see that I am worried. Because when he sees that I’m worried he will be more worried.

Me: Cancer is not infectious.

Me: How did you raise money for the bills, as you mentioned earlier that money was a major problem. Did you get a loan, or did you receive support form church, friends etc?

Kindness: yes, we took a loan, brothers, nephews and children helped a lot. We have never sought support from church or friends. We also depend mostly on God.

Me: Please have you sought counselling service anywhere?

Kindness: No please, but there is this nurse from outside this country who encourages me a lot. Also, one of our pastors and one or two friends. But the nurse really tried for me, because she is a geriatric nurse outside so she was able to tell me some of the things I need to do for him.

Me: Please when you were told that dad has the condition, did the nurses sit you down to educate you on what to do at home?

Kindness:No Please. It was only on the medication administration but nothing else

Me: Please is there anything you would love to say or ask?

Kindness: I am glad that a study of such kind is being conducted. Because the truth is that mostly the caregivers of these people go through a lot. Another issue has to do with some of the nurses, some are very disrespectful. They must change their attitude

Me: thank you very much

The interview ended with prayer.

19/03/2019

PARTICIPANT: HOPE

Interview lasted for 25 minutes/32 secs

ME: Explanation was given again on the need/reason for the study and consent form was signed after Hope has agreed to go through the interview

ME: Please may I know your age

HOPE: XX years old

ME: May I know your level of education?

HOPE: Tertiary Level

ME: So currently are you working?

HOPE: Yes, I’m working as a teacher here in XX

ME: Are you able to go to work?

HOPE: Yes, the school is close to my house

ME: So, what is your relationship with the patient

HOPE: He is my Dad

ME: Are you a Christian?

HOPE: Yes, I'm a Catholic

ME: How long has your dad been living with this condition?

HOPE: Okay, actually I was in Accra last year October and my dad called me that he fell. For the past, I think 6 months he has been sitting in a wheelchair because of the prostate cancer so I came to cape coast to support his movements. That is the main reason why I am here.

ME: So, it has been 6 months and not up to a year?

HOPE: yes, 6 months

ME: So, before he was diagnosed what were the signs and symptoms? Was he having problems with urination?

HOPE: No, it was when he fell down

ME: Or you are not aware because you were in Accra at the time

ME: It was when he fell that you went to the hospital ….?

HOPE: Yes, we went to XX for an MRI, so the next day that we came was when he started complaining that he can not urinate.

ME: When you went to Accra, how long has it been.

HOPE: Last October thereabout. But I have forgotten the exact date?

ME: Oh okay, so that’s how the condition all came about. HOPE: yes. ME: And since then you have been caring for him? HOPE: yes. ME: ok

ME: Where is your mum? Do you have siblings? Where are they?

HOPE: My mum is around and my other siblings are not here. I am the last born.

ME: Oh, so you have to stay and take care of your dad

HOPE: Yes, please

ME: So what exactly do you do for him daily?

HOPE: I help him take his bath to wear his shirt and trousers and everything.

ME: What about eating?

HOPE: He can eat by himself. So, I only put him in the wheelchair

ME: Who cooks for him?

HOPE: My mum cooks for him

ME: So, you also bring him for reviews and other things

HOPE: Yes

ME: Again, if I may ask. As a result of you taking care of him, can you say it has affected you physically? If I talk about physically, I mean you being able to have enough sleep, even your eating habits. For instance, your being here this morning you might not be able to make time to eat.

HOPE: Initially it was very difficult, especially carrying him here and there. But right now I’ve kind of acclimatized to the situation.

ME: You mean you are adapting gradually to it.

HOPE: Yeah

ME: So, tell me what you were experiencing before. I know you are still adapting to the situation but tell me to mean what u mean when you said it was difficult

HOPE: Ok, I was experiencing back pains, bodily pains all over.

ME: Were you getting tired most of the time?

HOPE: yes, but I didn’t want him to see that this is what I was going through

ME: Oh ok

HOPE: So even when I’m in pain, I don't complain

ME: So, you just want him to feel comfortable. HOPE: Yes

ME: Are you able to sleep?

HOPE: hmmm, sometimes

ME: Why are you not able to sleep?

HOPE: Because sometimes he complains of feeling pains in this penis. So, I have to wake up and give him antibiotics and pain killers that were given. When he is better before I can go to bed.

ME: Do you sleep in the same room with your dad?

HOPE: No, but my room is close to his room

ME: So psychologically, how has the role affected you. I hope you know that the role you are currently playing is partly the responsibility of the nurse?

HOPE: Initially my siblings decided to hire someone to assist me but I told them that I can do the job because I know what my dad has dome for me so I don’t complain

ME: oh ok, but then has there been a time when he has complained about something and you don’t know what to do?

HOPE: Yes, there have been times like that, but any time we find ourselves in a situation like that we have a family doctor that we call and in the next few minutes he will be in our house to come and take care of him.

ME: Do you sometimes get worried about what tomorrow holds as far as his current state is a concern?

HOPE: I have the belief that he will be fine in the next three months

ME: Do you sometimes feel helpless?

HOPE: No

ME: What about your social life

HOPE: As for me I like playing video games, so, when I’m free I play the game in my room

ME: Do you have friends?

HOPE: Yes, I do, I go to watch football with my friends. When my dad needs me, he calls my phone. My mum is also around.

ME: This means that you can not go far from home. You need to always be within reach because your dad can call at any time.

ME: Can I say that you had more time for yourself, and friends then as compared to now?

HOPE: yes, I’m not able to live my life the way I use to live it before. For instance, those days when I go out, I don’t come back home early but now I don’t spend more than 2 hours outside because I know that I will be called at home.

ME: Are you still in contact with most of your friends? Do you have a girlfriend?

HOPE: Yes

ME: Are you able to make time for her, or she understands the situation. Tell me about the state of your relationship then and now

HOPE: She is not here in XX. She also knows exactly what I’m going through.

ME: Where exactly is she?

HOPE: She is in Kumasi, she sometimes comes around

ME: okay, but are you able to visit her?

HOPE: No, and she understands.

ME: So, what are some of the things that made the role you play difficult?

HOPE: So, as I said before we have a family doctor who assists us in case of difficulty. If I’m not sure about something or don’t understand something I call him and he takes his time to take me through.

ME: What about the health workers here in the hospital. I mean the nurses especially. Has anyone taken the pain to educate you on what exactly the problem is, what you have done at home as a caregiver both at the OPD and when daddy was admitted?

HOPE: No one but what I do is I read around the condition and also on the medications. Apart from consulting our family doctor

ME: So, tell me what came to mind when you heard that your dad has cancer.

HOPE: Actually, I was not surprised even though I haven’t seen a catheter before.

ME: So, you mean even though you haven’t seen a catheter before and also seen it being used on someone you were not worried.

HOPE: yes

ME: Why?

HOPE: Because I knew he will be fine

ME: So, in terms of footing the bills, buying medications, running requested investigations and taking care of his overall needs, how do you and the family manage since dad and mum do not work anymore.

HOPE: Hmm, my siblings are the ones who support and sometimes my dad’s friends too. My siblings are five, plus myself we are six. The five are all working.

ME: What about loans?

HOPE: Not that I know of

ME: At the beginning, you said it was very difficult. Tell me more about.

HOPE: Yes, because I was preparing to write XX exams but because of this I had to put all my dreams, goals, and plans on hold. So, it was not easy at all to finally come up with the decision of putting everything on hold

ME: So, who did you speak to for you to be able to come up with that decision within a short time. Who advised and encouraged you? Is it your pastor, girlfriend, siblings, counsellors etc. What about him, do you go to church with him

HOPE: Nooo, he hasn’t been to church in the last six months but because we are Catholics that pastor comes home to give him communion and also pray for him.

ME: Earlier on you mentioned that you have been able to adapt to the situation, if not fullest partly. So, can you tell me some of the things you did that has helped you to adopt? Do you engage in active planning? For instance, planning your day to be able to make some time available for you

HOPE: I still live my normal life. Yes, I do plan my day but my mother is also around

ME: Do you take alcohol or drugs to help you to sleep or to release the stress sometimes

HOPE: Oh, not at all, even though I use to drink but in school, I don't drink since I came home

ME: Is there anything you would love to tell me or ay question you would love to ask?

HOPE: I don’t have a question but I want to say that this study you are conducting is very important because most caregivers do not know how to care for their relatives since it will be of help to them.

19/03/2019

PARTICIPANTS: FAITH

Interview lasted for 18 minutes/53 secs

Explanations have been given on the need/relevance of the study. Anonymity assured after which the consent form was signed

ME: Please what do you do for a living

FAITH: I am a XX, I sell banana

ME: Are you a Christian/Moslem?

FAITH: Christian

ME: Who is the patient to you?

FAITH: My father

ME: Please tell me everything that has transpired since your dad was diagnosed. All that you have been through since you started playing the role of the caregiver.

FAITH: When we were first told about the condition and treatment. It got me very scare and worried especially about the cost of treatment. After doing all the laboratory investigations requested, the doctor said every month we will have to pay 400 GH cedis for medications and 200gh cedis for injections, making a total of 600gh cedis per month. I know that it will be very difficult to raise 600gh cedis every month. What even made matters worse was when we were told that this 600gh cedi will be paid every month for 2 years. The doctor then made a total sum of the entire treatment for the 2 years and it was almost 15000 GH cedis. This was very worrying. because as said earlier, I am a petty trader, how on earth can we raise such an amount. But finally, we went for a loan for the treatment.

ME: So, a loan has been your family source of financial support for your dad’s treatment?

FAITH: yes

ME: So, since he was diagnosed, you have been the caregiver all through?

FAITH: Yes, but when it comes to financial needs, I call my other siblings.

ME: So, who takes care of his hygiene needs as well as nutritional needs?

FAITH: I take care of everything except monetary aspects. Even that, I support with the little I have. But now the surgery has been done so we thank God because it means that we will have a less financial burden

ME: Looking at your current state of being heavily pregnant coupled with the stress of caring for your dad, can I say that, the role you are playing is greatly affecting you?

FAITH: Yes, but I try to endure because in this state If I worry too much I may even die and leave him. So, I try as much as possible not to worry too much. However, he is rather the one who worries a lot. I try to encourage him by making him understand that everything will be fine and not need to be afraid.

Most often I bring him to see the doctor when the date of review is not even up, because he continuously complains about pain everywhere. But I thank God for how far he has brought us in this journey because it hasn’t been easy.

ME: How does your current role affect your sleep. Also, eating pattern. You told me you haven't had anything this morning and it is almost noon.

FAITH: Yes, mostly I want him to eat something and recover quickly. I’m not worried about me but more worried about him. Because I believe that if he is okay I will also be fine.

ME: wow

ME: I know that if one is very worried, it can affect one’s sleep. So since you are worried most often are you able to sleep?

FAITH: I’m not able to sleep at all, especially when the old man is in so much pain and we were not receiving any feedback from the hospital and doctors as to when the surgery will be done. It was worrying because I also thought that probably the doctor has seen something that may result in an unexpected occurs during the surgery thus the reason we haven’t been called. I had the contact of one of the doctors so I called and he told me that sometimes cases are booked and for months they still haven’t been able to do them so it is not a problem. In fact, after calling the doctor I was quiet relief.

ME: Please were you told about the condition?

FAITH: yes

ME: What were you told and who told you?

FAITH: the doctors told that a disease has been seen in his manhood, which is cancer so he will need treatment then subsequently surgery.

ME: So, what was your reaction when you heard that your father has cancer?

FAITH: hmm, my heart skipped a beat when I heard he has cancer

ME: Why?

FAITH: Because I’ve heard that cancer kills, but later when the doctor explained everything to us, I became less worried

ME: Do you sometimes feel overwhelmed to the extent that you even want to give up on everything?

FAITH: Yes, because what happens is, my father, complains a lot, and will insist that I bring him over to see the doctor even when the review date is not up. When we come the doctor will tell us there is nothing wrong with him/not sick, sometimes we will even spend money to do other investigations based on the complains he presents to the doctor. But the results come out negative.

ME: So, tell exactly what the doctors told you.

FAITH: They told us that if we can do the treatment and surgery it will increase his life expectancy for another XX years and more. Currently, he is XX yrs.

ME: please are you able to make time for friends, programmes and what about your immediate family.

FAITH: As for attending programmes and spending time with friends its impossible for now because I need to make sure my dad is okay and I also can’t leave him and go anywhere.

ME: But what about your friends don’t they complain?

FAITH: Not really. Some of them even pass by to find out how he is doing.

ME: So, what about your immediate family. I mean your children and husband? How many children do you have including the unborn baby? How old is the eldest?

FAITH: I have 6 children, plus the one I’m carrying now they will be 7. The eldest is XX yrs. My husband is very supportive and he understands the situation. He mostly takes care of the children when I’m not around so I don’t have any problems in that regards. My eldest child also assists in so many ways. She can take care of her siblings in our absence.

ME: So, since your dad was diagnosed with the condition, has any of the nurses or doctors taken the time to sit you down to explain to you how you will have to care for him at home, what to do, what not to do etc.

FAITH: The doctor we first met was a woman, she then referred us to a different doctor. So the doctor we were referred to was the one who told me to be patient with him because now he is old and with a condition like this it will be very difficult for the both of us.

ME: What about the nurses you met when your dad was on the ward?

FAITH: There was a particular nurse who assisted us. She was very nice to us. Even, before I got here on the day of surgery, she had prepared my dad and already sent him to the theatre.

ME: Earlier on you mention that you do all u can to encourage your sick dad through this difficult time. Who encourages you, since you are in this with him?

FAITH: My other siblings do, especially when I call them for money and I also encourage myself all the time. Now I have come to terms with the situation hence left every personal thing I’m doing and devoted myself in taking care of him. I also believe that the lord can heal him, so that has been my prayer anytime I go to church. But as for him, he wouldn't want to go to church. So, I go and pray for him.

ME: Do you have any question for me?

FAITH: Not, please

ME: Please is there anything you would like to tell us

FAITH: Yes, please I would like to plead with the ward and the hospital that if there is anything that needs to be done for our patients in relation to their treatment, they should do it so that when the relatives come around they can pay for them rather than leave the patients in pain when the relatives have been sent out of the unit
